# Biophysics in tumor growth and progression: from single mechano-sensitive molecules to mechanomedicine

**DOI:** 10.1038/s41388-023-02844-x

**Published:** 2023-10-20

**Authors:** Ying Xin, Keming Li, Miao Huang, Chenyu Liang, Dietmar Siemann, Lizi Wu, Youhua Tan, Xin Tang

**Affiliations:** 1 The Hong Kong Polytechnic University Shenzhen Research Institute, Shenzhen, China; 2 Department of Mechanical and Aerospace Engineering, Herbert Wertheim College of Engineering, University of Florida, Gainesville, FL USA; 3 UF Health Cancer Center, University of Florida, Gainesville, FL USA; 4 Department of Biomedical Engineering, The Hong Kong Polytechnic University, Hong Kong, China; 5 Research Institute of Smart Ageing, The Hong Kong Polytechnic University, Hong Kong, China; 6 J. Crayton Pruitt Family Department of Biomedical Engineering, University of Florida, Gainesville, FL USA; 7 Department of Physiology and Functional Genomics, University of Florida, Gainesville, FL USA

**Keywords:** Biophysics, Cancer

## Abstract

Evidence from physical sciences in oncology increasingly suggests that the interplay between the biophysical tumor microenvironment and genetic regulation has significant impact on tumor progression. Especially, tumor cells and the associated stromal cells not only alter their own cytoskeleton and physical properties but also remodel the microenvironment with anomalous physical properties. Together, these altered mechano-omics of tumor tissues and their constituents fundamentally shift the mechanotransduction paradigms in tumorous and stromal cells and activate oncogenic signaling within the neoplastic niche to facilitate tumor progression. However, current findings on tumor biophysics are limited, scattered, and often contradictory in multiple contexts. Systematic understanding of how biophysical cues influence tumor pathophysiology is still lacking. This review discusses recent different schools of findings in tumor biophysics that have arisen from multi-scale mechanobiology and the cutting-edge technologies. These findings range from the molecular and cellular to the whole tissue level and feature functional crosstalk between mechanotransduction and oncogenic signaling. We highlight the potential of these anomalous physical alterations as new therapeutic targets for cancer mechanomedicine. This framework reconciles opposing opinions in the field, proposes new directions for future cancer research, and conceptualizes novel mechanomedicine landscape to overcome the inherent shortcomings of conventional cancer diagnosis and therapies.

## Introduction

Cancer accounts for 12.6% of all deaths worldwide and 90% of cancer-related deaths are due to metastasis: the dissemination of tumor cells from the primary neoplastic lesion to other organs [1]. Hence, identifying key regulators in the formation and progression of the primary tumor and metastases is crucial to the prevention, prediction, early diagnosis, and treatment of cancer. Past decades have witnessed the growing significance of the reciprocal interactions between tumor cells and their microenvironment in the primary tumor and metastatic process. The biochemical and genetic signals involved have been intensively investigated. Besides these chemical-genetic factors, burgeoning evidence has clearly demonstrated that mechanical cues arising from the alterations in the biophysical properties of tumor cells, tissues, and microenvironment also substantially contribute to this dynamic reciprocity and eventually influence tumor growth and progression.

Existing findings on tumor biophysics are limited, and vary and at times appear contradictory due to a variety of reasons, including those of a technical nature, limiting our systematic understanding of the roles of biophysics in tumor pathophysiology. Further, the biophysical mechanisms, especially mechanotransduction signaling, as well as their crosstalk with classical oncogenic pathways are still poorly understood. Many findings on tumor mechanobiology have been discussed in several excellent reviews [2–5]. However, few have (1) offered an analysis of the “seemingly contradictory” findings and (2) discussed the emerging mechano-medicine as a possible route to transform the future of cancer therapeutics. Therefore, we undertake a comprehensive and objective review of literature, aiming to clarify and reconcile contradictory findings in the field and to sort out new directions for cancer treatment. First, we discuss the altered mechano-omics of the primary tumor tissue and cells within neoplastic niche. Second, we address the functional crosstalk between mechanotransduction and oncogenic signaling. Third, we highlight the recent advance in multi-scale technologies for the study of cancer mechanobiology and mechano-medicine that holds potential as promising anti-cancer strategies. We conclude by summarizing the reconciled hypotheses and providing new perspectives on future directions of tumor mechanobiology and mechano-therapeutics.

## Mechanics in tumor growth

### Mechanical alterations in primary tumor tissue

#### Tumor tissue mechanics

**Fig. 1:**
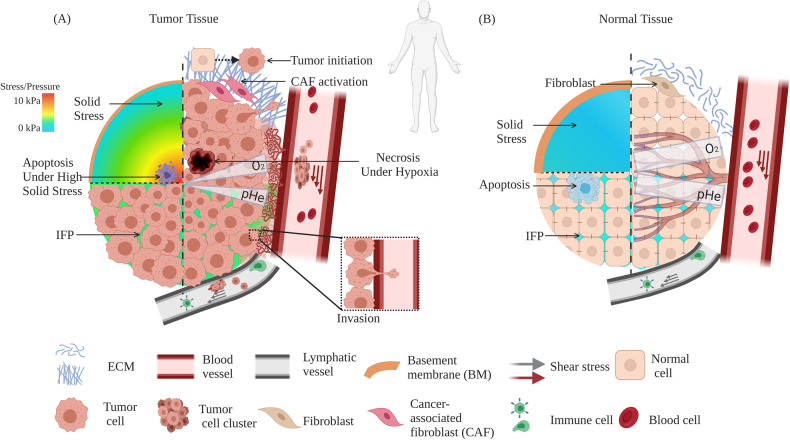
Biophysical alterations in primary tumors. The cross-sections of tumor tissue (**A**) and normal tissue (**B**) are shown in cartoons, respectively. Cells within tumor tissues experience solid mechanical stress from the structural components and interstitial fluid pressure (IFP). The upper-left quarter of (**A**) and (**B**) shows the solid stress distribution. The left-bottom quarter shows the IFP distribution. Tumor tissue has higher solid stress with gradient (decrease from the tumor core to the tumor periphery) and higher IFP than those in normal tissue. High solid stress (4–15 mmHg) facilitates tumor cell motility but solid stress that is higher than 37 mmHg induces tumor cell apoptosis. The right half of (**A**) and (**B**) shows the constituting cells and the mechanical/chemical stimuli within the microenvironment. Tumor cells have abnormal morphology and disorganized structures compared to normal cells. ECM in a tumor is stiffer, denser with more crosslinking, and aligns more perpendicular to the tumor boundary compared to the loose and isotropic ECM in normal tissue. The altered ECM facilitates tumor initiation, invasion, migration, metastasis and CAF activation. Higher shear flow in tumor interstitial fluid increases tumor cell invasiveness and motility. Tumor cells can escape apoptosis while normal cells cannot. Blood vessels in the tumor form dendritic and leaky structure that only reaches the tumor periphery due to high confinement from tumor core. Hypoxia caused by these abnormal blood vessels in the tumor causes abnormal gradient of extracellular pH (pHe) and necrosis of tumor cells. In contrast, blood vessels in normal tissue can penetrate through the tissue and produce normal oxygen/pHe levels.

**Fig. 2:**
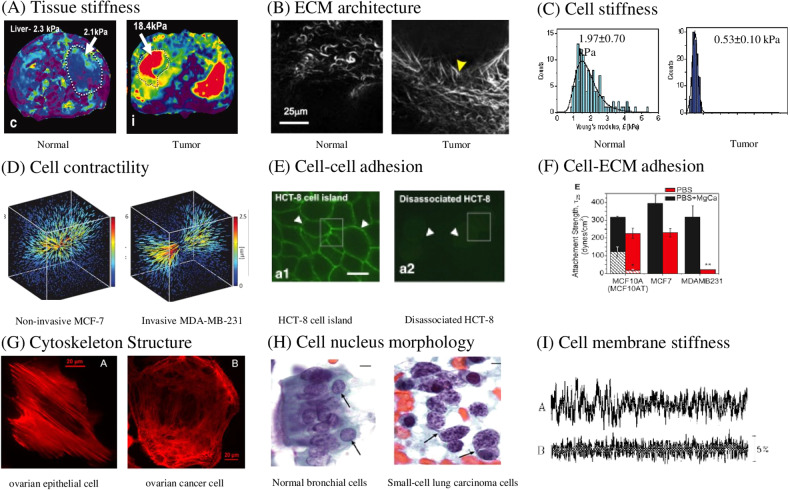
Comparison of mechanical properties between normal tissue/cells and tumor tissue/cells or between tumors with different levels of malignancy. **A** Magnetic resonance elastography (MRE) imaging and measurement of tumorous and normal liver tissues. Malignant tumor tissue is mechanically stiffer than normal tissue [435]. **B** Second harmonic generation (SHG) imaging shows crosslinking ECM in tumor tissues and loose ECM in normal tissues [53]. **C** Tumor cells show lower mechanical stiffness than normal cells [109]. **D** Higher and more polarized contractility (demonstrated by magnitude and local concentration of traction forces) in invasive breast cancer cells MDA-MB-231 and lower and nonpolarized traction force in noninvasive MCF-7 breast cancer cells [176]. **E** Cell-cell adhesion (indicated by expression amount and distribution of cell adhesion molecule E-cadherin) is lower in disassociated colon cancer HCT-8 cells than in nondissociated HCT-8 cell island [436]. **F** Invasive MDA-MB-231 breast cancer cells show lower cell-ECM adhesion than noninvasive MCF-7 breast cancer cells [165]. **G** Ovarian epithelial cells’ actin cytoskeleton contains linearized F-actin, while ovarian cancer cells’ cytoskeleton shows crosslinking F-actin [112]. **H** Normal bronchial cells show round nucleus (purple) with finely granular heterochromatin (dark-violet) while small-cell lung carcinoma cells show elongated or random shaped nucleus (purple) with clumping chromatin (dark-violet) [188]. **I** Highly metastatic BW-19 lymphoma cells (**A**) have higher cell membrane stiffness than low metastatic BW-19cl1 cells (**B**) [205].

Solid tumors and their associated tumor microenvironment (TME) consist of tumor cells and stromal components, including extracellular matrix (ECM), basement membrane (BM), vasculature, immune cells, and fibroblasts (Fig. 1A). During tumor progression, all components change their physical structures and functions [6–8]. With few exceptions, primary tumors in many cancer types are usually more mechanically rigid than their healthy tissues of origin (Figs. 1B and 2A) [2, 6, 9]. For example, human breast tumors are 5-fold stiffer than healthy tissues and such high stiffness positively correlates with malignancy [10]. Mouse tumor mammary tissue is 24-fold stiffer than healthy mammary tissue [11]. Human liver tissue stiffness positively correlates with the risk of hepatocellular carcinoma with a cut-off value at 20 kPa [12]. Besides overall stiffening, another salient mechanical hallmark of tumor tissue is the heterogeneity of intratumoral stiffness [13]. The measurement by ultrasound elastography shows the considerable spatial variation of tissue stiffness in breast and liver tumors [14]. In human breast tumor biopsies, the tumor periphery is 7-fold stiffer (E = 5.51 ± 1.70 kPa) than the tumor core (E = 0.74 ± 0.26 kPa), while healthy breast tissue stiffness is 1.13–1.83 kPa [15]. Other than stiffness, the visco-elasticity of tumor tissues also differs from that of normal tissues. For example, the in vivo measurement by magnetic resonance elastography (MRE) shows that the fluidity of human benign meningioma tissue is 3.6-fold higher than aggressive glioblastoma tissue. This solid-like behavior of glioblastomas facilitates its aggressive penetration through the surrounding tissue [16].

The elevation in the tumor tissue stiffness is mainly due to excessive deposition and enhanced crosslinking of ECM, especially collagen (Fig. 2B) [17]. Remodeled continuously by tumorous and stromal cells, the TME provides physical-chemical cues to regulate gene expression and functions of these cells by activating a number of intra- and extracellular molecular receptors and signaling pathways, such as integrin, PIEZO 1/2, and Rho/ROCK. These receptors sense and transduce extracellular biophysical signals into the nucleus, followed by relaying intracellular feedback to remodeling extracellular TME [18, 19]. The composition, stiffness, and architecture of ECM determine its regulatory roles in tumor progression. ECM is composed of fibrous proteins, glycoproteins, proteoglycans, and polysaccharides [20]. High expressions of various ECM proteins are correlated with poor prognosis in many types of cancer [1, 17]. Abnormal expressions of ECM enzymes, e.g., matrix metalloproteinases (MMPs) that regulate ECM remodeling, are considered as indicators of poor prognosis [18]. As the main structural components of ECM, collagens contribute to up to 60% of the tumor mass and tumor tissue stiffness [19, 20]. High collagen density promotes the initiation of breast tumor and invasive phenotypes [21]. ECM stiffness critically influences tumor cell transformation, proliferation, and motility. For example, high ECM stiffness facilitates YAP nuclear localization, which is required for the RTK-Ras oncogene induced transformation of normal breast cells [22]. Breast cancer cells express a higher level of miR-18a on stiffer ECM, which facilitates tumor cell growth [23]. Stiff ECM promotes the growth and invasion of breast tumor cells by inducing high cell tension. High ECM stiffness upregulates TWIST1, which promotes epithelial-mesenchymal transition (EMT) and the metastasis of breast cancer cells [24]. In pancreatic ductal cancer cells, high ECM stiffness activates a signal transducer and activator of the transcription 3 (STAT3) pathway, which increases matricellular fibrosis and ductal epithelial tension, and promotes tumor progression through reduced TGF-β signaling and increased activation of β1-integrin [25]. High ECM stiffness and cell contractility increase MMP activity of pancreatic cancer cells by 3–10-fold, which enhances the migration, invasion, and angiogenesis [26]. The spatial distribution of liver cancer stem cells (CSCs) correlates with tumor tissue stiffness: tumor periphery is 13-fold stiffer and contains 13-fold more CSCs than the tumor core [27]. In response to high ECM stiffness, glioma cells activate Piezo1 at focal adhesion sites and increase calcium influx, which activates integrin-FAK signaling and reinforces ECM stiffening [28]. High tissue stiffness activates the Rho/ROCK pathway to increase actomyosin-mediated cellular tension and collagen deposition that in turn enhance tissue stiffness [29]. Further, tumor tissue stiffness influences vascular morphology, barrier function, and permeability [30–33]. For example, matrix stiffness-induced FAK activity activates Src and high levels of phosphorylated vascular endothelial cadherin (VE-cadherin) at adherent junctions of endothelial cells [30]. Increased ECM stiffness results in more angiogenic sprouting and permeability, undesirably enhancing the spread of tumor cells into the vasculature [31]. Although both density and crosslinking level of collagens contribute to ECM stiffness, they can oppositely influence angiogenesis. In an in vitro 3D organ culture model of sprouting angiogenesis, increased matrix density reduces angiogenesis and vessel network formation, possibly because stiff ECM is harder for endothelial cells to deform [34]. Increased collagen crosslinking promotes the angiogenic sprouting of the spheroid and increases substrate stiffness [31]. Notably, thicker and more linearized ECM is observed in the region adjacent to tumor vasculature [35]. These findings suggest that the influence of the increased ECM density and alignment (crosslinking and linearization) on tumor stiffening and progression could be independent from each other. The effect of ECM stiffness on angiogenesis is dependent on cell-ECM adhesion. On 2D collagen-coated polyacrylamide (PA) gels, softer ECM (200 Pa vs. 10 kPa) promotes the formation of an endothelial cell loop that mimics the angiogenesis initiation, but switches to the suppressive effect when the collagen density is reduced from 100 μg/mL to 1 μg/mL.

The effect of ECM stiffness on tumor progression is, however, somewhat controversial. For example, ovarian cancer cells are more invasive in softer environments [36]. Several reports show that low ECM stiffness maintains the stemness of malignant tumor repopulating cells (TRCs or CSCs) in a soft (90 Pa) but not stiff (1.05 kPa) ECM [37–39]. The compliance of a magnetic platform with high ligand tether mobility upregulates the stemness and tumorigenicity of tumor cells [40]. The CD133+ liver CSCs soften local niches to maintain their stemness, enhance drug resistance, and facilitate metastasis [41]. These distinct responses to ECM stiffness may be due to the dependence of mechanosensing on specific cancer type as well as the heterogenous TME and tumor cell subpopulations. As a fibrous material, collagen shows the traits of strain hardening, nonlinear elasticity, and anisotropy [42, 43]. ECM stiffening can be caused by collagen strain hardening even at low cell-contraction-induced strain and reciprocally facilitates tumor progression if the strain hardening is irreversible [44, 45]. In a finite element model, the nonlinearity of collagen fibers (compression buckling and tension stiffening) is found to facilitate the long-distance (~9-cell-length) transmission of mechanical signals to distant cells [46]. Besides stiffness, ECM architecture, e.g., fiber alignment, crosslinking, porosity, and topography, also mediates the invasive phenotypes of cancer cells, including motility, protrusions, and MMP activity in self-assembled 3D collagen matrices [47, 48]. High collagen density can reduce ECM pore size and a moderate pore size (5–12 μm) is considered a key promoter for glioma invasion [49]. By exploiting an interpenetrating network of hydrogels (from 30 Pa to 310 Pa), the effects of pore size and stiffness on cancer cells have been decoupled [50]. The confinement in the pores enhances the polarization, traction force, and migration speed of cancer cells. Cell migration speed is positively related to ECM stiffness in the spatially confined ECM, while this relationship is biphasic in unconfined ECM [51]. In breast cancer, collagen fibers that align perpendicularly to the tumor boundary are found to promote invasion and metastasis [52]. High levels of collagen crosslinking, together with increased ECM stiffness, facilitate tumor cell invasion by enhancing integrin-regulated FAK-Src signaling [53]. ECM in the TME is mainly produced by stromal cells, including cancer-associated fibroblasts (CAFs). Multiple pathophysiological changes can alter the number and function of CAFs to facilitate tumor progression. For example, obesity enhances the local population of myofibroblasts in mammary adipose tissue, increases interstitial fibrosis and ECM stiffness, and promotes tumorigenesis [54]. Stiff ECM may maintain CAF phenotype and reinforce ECM stiffening through two potential mechanisms. First, in primary rat lung fibroblasts, stiff ECM and high tissue tension are necessary for the generation of contractile forces that can stretch the large latent complex (LLC). TGF-β stored in LLC is released upon stretching and converts fibroblasts into myofibroblasts [55]. Second, stiff substrates enhance YAP activity and contractility in fibroblasts that are required for CAFs to promote matrix stiffening, cancer cell invasion, and angiogenesis [56]. A feed-forward self-reinforcing loop has been reported between YAP activation, Src function, and cytoskeleton contractility to generate and maintain CAF phenotypes [57]. However, CAFs show either pro- or anti-tumor effects. On one hand, through producing ECM components, CAFs can increase tumor’s adaptive resistance to chemotherapy through influencing ECM structure and mechanics, reprogramming cancer cell metabolism, and changing the immune responses [58, 59]. On the other hand, for PDACs in mice/patients, depletion of alpha smooth muscle actin (αSMA)+ myofibroblasts leads to invasive and undifferentiated tumors with enhanced hypoxia, EMT, and CSCs that likely deteriorate subject survival [60]. These studies suggest that further understanding of CAF’s roles in tumors is demanded and caution must be taken when targeting CAFs in cancer therapies.

#### Basement membrane mechanics

As a thin (20 nm–10 µm thickness) and porous (10 nm–112 nm pore size) crosslinked ECM sheet, BM mainly consists of collagen IV and laminin, separates the tumor tissue from the surrounding normal/stromal tissue, and forms the outer boundary of blood vessels [61, 62]. Compared to the healthy BM, the tumor BM contains less collagen IV and laminin and is thinner, more discontinuous, and less crosslinked [63, 64]. The weakened integrity of the BM is a major hallmark of multiple cancer types [65]. Interestingly, the BM is much stiffer in many tumor tissues (~10 kPa for colon, skin and breast cancer) than in normal ones (~3 kPa for breast glands and ~0.12 kPa for prostate glands) [66–70]. However, the actual mechanisms remain unclear.

Invading tumor cells must physically breach through BMs during metastasis [71]. Therefore, the mechanical properties and composition of the BM critically impact tumor cell invasion [72, 73]. For example, Net4-mediated BM softening (25 kPa compared to 50 kPa) reduces the invasion of mouse breast cancer cells even though Net4 results in larger pore size [73]. Increased stiffness of BM-like substrates reduces the aggregation of integrin α6β4 and exposes its multiple sites for phosphorylation by receptor tyrosine kinase (RTK), leading to activation of PI3K and Rac1 signaling, which induces a malignant phenotype [50]. However, another study reports that reducing the BM stiffness by targeting Col4a1 enhances tumor invasion in the mouse model [74]. The stiffness and composition of the BM work together to regulate cancer cell invasiveness [50]. A rigid BM induces the invasion of MCF-10A, while the increase in laminin density stiffens the BM and inhibits normal breast cell cluster invasion, suggesting the complex roles of BM composition and stiffness in tumor cell invasion [75]. Thus, it is unclear whether targeting Col4a1 affects tumor cell invasion through the effect on BM stiffness or composition. Other than Young’s modulus, the plasticity (the ability of a material to permanently retain deformation) of the BM also influences tumor cell behavior. For example, covalent cross-linking between a reconstituted BM and tissue transglutaminase reduces the plasticity of the BM but maintains similar Young’s modulus. The BM with slow stress relaxation suppresses the spreading and protrusion formation of breast cancer cells [76].

Cells can invade the BM in both protease-driven and force-driven modes. High stiffness of collagen I matrix can increase MMP activity at the locations of cancer cell protrusions to degrade ECM and induce invasion [48]. However, whether this is applicable to the BM that contains mainly laminin and collagen IV is not clear. During angiogenesis, tumor cells degrade the existing BM by proteases and facilitate initial laminin polymerization through surface proteins and BM assembly [45]. This remodeling process may create leaky blood vessels in the tumor and contribute to the onset of metastasis [46, 72]. In the force-driven mode, CAFs generate mechanical force to enlarge the preexisting gap in the BM to 6.2 ± 1.7 µm in diameter and soften the BM, which facilitate cancer cell invasion in an MMP-independent manner [77].

Tumor cells can sense mechanical tension in vitro and potentially the increased BM tension in vivo. One important, but currently unknown question is how BM tension, that is related to tumor growth physically, contributes to the weakening of BM and modulates downstream signaling in BM-adjacent tumor cells.

#### Solid stress

In a solid tumor, intratumoral residual or solid stress builds up due to physical resistance from the surrounding healthy tissue against the outgrowth of tumor cells (Fig. 1A) [78]. Within the same tumor in melanoma, mammary adenocarcinoma, and breast cancer, solid stress increases as the tumor grows, suggesting that increased solid stress may be a mechanical hallmark of cancer [44, 79, 80]. Nevertheless, the magnitude of solid stress is not proportional to tumor volume across different cancer types [44].

Solid stress affects multiple tumor cell functions (Table 1). Solid stress inside a colon tumor (1.2 kPa or 9 mmHg) activates the oncogenic β-catenin pathway in the surrounding healthy tissue, which facilitates the generation of hyper-proliferative crypts independent of tumor tissue stiffness [81]. Solid stress at 0.53–2 kPa (4–15 mmHg) enhances breast/pancreatic/renal cancer cell motility [81–85]. However, once the solid stress exceeds a certain threshold (>37mmHg), it suppresses the growth of tumor spheroids and triggers cell apoptosis [86–88]. Within the same colon tumor spheroids under isotopic 5 kPa (37.5 mm Hg) pressure, cells at the periphery experience lower levels of pressure (1 kPa, 7.5 mmHg) and proliferate faster than those in the core that experience higher levels of pressure (8 kPa, 60mmHg) [89]. These findings suggest that the intratumoral solid stress of different magnitudes at different stages of tumor growth seems to influence tumor cell functions distinctly. Apart from its influences on tumor cells, solid stress at 0.53 kPa (4.0 mmHg) activates fibroblasts, which further facilitates pancreatic cancer cell migration [90]. In vivo application of compressive force induces vascular perfusion deficiency and neuronal damage. When the compressive stress is removed, both the neuron function and motor coordination restore [91].

**Table 1:** Distinct influences of solid stress on tumor cell functions.

Magnitude and direction of solid stress	Effects on tumor progression	Cell types	Mechanisms	Refs
4 mmHg (Compressive)	Enhance cancer cell motility	Renal cancer cells (monolayer; in vitro)	Activate Akt/GSK-3β/β-catenin signaling pathway	[83]
4 mmHg (Compressive)	Promote tumor cell migration	Pancreatic cancer cells (monolayer; in vitro)	Activate Akt/CREB1 pathway	[84]
5.8 mmHg (Compressive)	Promote mammary carcinoma cell migration and adhesion	Breast cancer cells (monolayer; in vitro)	Enable the formation of leader cells and elevate cell-substrate adhesion	[85]
9 mmHg (Compressive)	Enable oncogene activation and transform normal tissues into cancerous tissues	Healthy colon tissue (mouse)	Activate Ret and the downstream phosphorylation of β-catenin	[81]
15 mmHg (Compressive)	Increase the motility of peripheral cells	Mouse colon carcinoma cells (multicellular spheroid)	NA	[82]
37.5-75 mmHg (Compressive)	Inhibit multicellular tumor spheroid proliferation	Colon carcinoma cells; human breast cancer cells; mouse sarcoma cells (multicellular spheroid)	Induce the expression of the proliferation inhibitor p27Kip1	[87]
60 mmHg (Compressive)	Suppress cell proliferation and induce apoptosis	Murine mammary carcinoma cells (multicellular spheroid)	Increase caspase-3 activity	[86]
45–120 mm Hg (Compressive)	Inhibit the growth of multicellular tumor spheroids	Human colon adenocarcinoma cells; Murine mammary carcinoma; Rat rhabdosarcoma cells (multicellular spheroid)	NA	[88]

Almost all current studies have utilized compressive stress to represent solid stress. However, the direction of solid stress varies at different intratumoral locations, e.g., tensile stress at the periphery and compressive stress in the interior [44]. Hence, further studies are needed to elucidate the influence of tensile solid stress on tumor malignancy. In most studies, solid stress has been applied in vitro and the influence of the surrounding normal tissue is ignored. Some studies have shown in the same tumor that the maximum compressive solid stress is 0.02 kPa (0.15 mmHg) in the ex vivo measurement while reaching 0.1 kPa (0.75 mmHg) in the in-situ measurement, which may be attributed to the difference in the surrounding microenvironment [79]. Therefore, developing new methods that can exert solid stress in vivo is important to study the influence of solid stress on tumor progression and provide physiologically relevant insights [81, 92].

In addition to the direct effects on tumor cell functions, the intratumoral solid stress can influence tumor progression indirectly by compressing blood and lymphatic vessels, inducing hypoxia, and suppressing nutrient transportation and therapeutic delivery [93, 94]. The underlying mechanisms and therapeutic treatments are detailed in “Mechano-medicine and mecjanotherapy” seection. It is possible that the compression of blood and lymphatic vessels by solid stress impacts the distribution of nutrients and metabolites within the tumor in vivo and tumor spheroids in vitro, influencing tumor cell functions. However, most current studies have not considered this indirect effect. The influence of hypoxia can be neglected for the in vitro cell monolayer and tumor spheroids with radius less than 200 μm because their size is within the oxygen diffusion limit (400 μm) [95, 96]. However, when the 3D tumor size exceeds this limit, the influence of stress-induced hypoxia should be taken into consideration [87, 88].

#### Interstitial fluid pressure (IFP) and interstitial flow

Interstitial fluid is the body fluid in the tissue stroma (Fig. 1A). The IFP is elevated from –3-3 mmHg in healthy tissues to 10–100 mmHg in tumor tissues due to the irregular structure and elevated permeability of vasculature, disordered blood flow, stiffened interstitial matrix, and the impaired drainage function of lymphatic vessels [97–99]. High IFP is closely associated with poor prognosis and survival of cancer patients. Lung cancer patients with IFP >7.4 mmHg have lower recurrence-free survival (RFS) than patients with lower IFP [100]. Chemotherapy treatment lowers IFP in patients with rectal cancer (from 15.0 ± 2.0 mmHg to 4.0 ± 2.2 mmHg) and breast cancer (from 7.0 ± 1.9 mmHg to 4.4 ± 1.8 mmHg) and leads to anti-vascular and increased oxygenation effect [101, 102]. After radiotherapy, IFP higher than 19 mmHg is correlated with lower disease-free-survival (DFS) of cervix cancer patients [103].

IFP influences tumor cellular functions and malignancy. The reduced IFP suppresses cell proliferation by decreasing the expressions of p44/42 MAPK and Ki-67 [104]. Increasing IFP from 0 mmHg to 15 mmHg and to 30 mmHg facilitates the proliferation and invasion of oral squamous carcinoma cells [105]. Hydrostatic pressure of 20 mmHg increases the volume of lung cancer cells through p-ERK and water-penetrating membrane channel AQP1 and enhances cell motility [106]. Apart from the static fluid pressure, the interstitial flow (0.1–50 µm/s in the tumor) and the resultant shear stress (around 0.1 dyne/cm2) also alter tumor and stromal cell functions [30, 31]. Fibroblasts, but not the TRPM7-deficient fibrosarcoma cells, reverse their migration direction under 0.5 dyne/cm2 shear flow [32]. 4.6 µm/s (16.8 pN shear force) interstitial flow reorganizes focal adhesion of breast cancer cells in 3D collagen and enhances cell migration [33]. In the 3D tumor-lymphatic-mimicking microenvironment, tumor cells that are exposed to 0.2 µm/s interstitial flow show higher motility [34].

#### Hypoxia

The outgrowth of tumor cells, the deteriorated microcirculation, and inadequate oxygen diffusion reduce the oxygen content from 4.6% to 9.5% in healthy tissues to < 1%–2% in tumor tissues, resulting in tissue hypoxia. Increasing tumor hypoxia is inversely correlated with clinical outcomes and patient survival [107].

Increasing evidence indicates the correlation between hypoxia and tumor tissue mechanics. Various types of tumor tissues show a lower level of oxygen content and higher bulk stiffness than their normal counterparts (Table 2). Apart from this correlation, hypoxia directly influences the mechanics and functions of tumor cells. In breast cancer cells, intratumoral hypoxia promotes the transcription of Rho family members RHOA and Rho-kinase 1 (ROCK1), and enhances the stress fiber formation, matrix contraction, and cell motility [62]. In hepatocellular carcinoma cells, hypoxia promotes actin remodeling via the HIF-1α/RhoA/ROCK1 pathway and initiates EMT to facilitate tumor invasion and migration [71]. In addition, hypoxia influences the mechanics of CAFs and tumor metastasis. Under 80-hr 2D culture in hypoxia (1% oxygen), CAFs produce a stiff and well-aligned layer of collagen I by inducing the expressions of the genes encoding collagen prolyl (P4HA1 and P4HA2) and lysyl. Further, hypoxia promotes actin remodeling in CAFs, leading to increased fibrillar collagen deposition, and promoting their pro-malignant functions [108]. Interestingly, in 3D collagen, the same level of hypoxia (1% oxygen, 72-hr) inhibits PHD2, stabilizes HIF-1α, reduces the expressions of alpha‐smooth muscle actin (αSMA) and myofibroblast modulator periostin, and decreases myosin II activity and the stiffness of CAFs, therefore deactivating CAFs. Deactivated CAFs reduce the ECM remodeling and tumor stiffness, and inhibit CAF-mediated lung metastases of breast cancer. These contradictory results may be due to the dimension differences of the experimental models.

**Table 2:** Comparison of the oxygenation and stiffness in tumor tissues and their healthy counterparts.

Cancer type and the healthy tissue	Physoxia (median % oxygen) in healthy tissue	Healthy tissue stiffness (measurement methods)	Hypoxia (median % oxygen) in tumor	Tumor tissue stiffness (measurement methods)	Refs
Brain	4.6%	7.3±2.1 kPa (normal brain tissue) (Shear wave elastography)	1.7%	33.1 ± 5.9 kPa (meningiomas), 23.7 ± 4.9 kPa (low-grade gliomas), 11.4 ± 3.6 kPa (high-grade gliomas), and 16.7 ± 2.5 kPa (metastatic tumor) (Shear wave elastography)	[443–446]
Breast	8.5% (a median pO2 value of 65 mmHg)	1.13~1.83 kPa (mouse breast tissue) (AFM)	1.5% (the median pO2 was 30 mmHg)	A bimodal distribution with two prominent peaks as 0.45±0.12 and 1.29±0.76 kPa (mouse breast tumor) (AFM)	[15, 443, 447]
Cervix (nullipara)	9.5% (a median pO2 value of 42 mmHg)	lower elasticity peak (8.51±0.18 kPa) and higher elasticity peak (44.07±3.54 kPa) (Human cervix tissue) (AFM)	1.2% (2~34 mmHg)	lower elasticity peak (LEP) (21.24±3.83 kPa) and higher elasticity peak (HEP) in the cancer group (81.23±8.82 kPa) (Human cervix tumor) (AFM)	[447–449]
Liver	4.0~7.3% (normal huamn liver tissue)	0.91 ± 0.44 to 1.46 ± 0.60 kPa (normal liver tissue) (AFM)	0.8% (liver tumor)	LEP (from 0.15 ± 0.13 to 1.05 ± 0.45 kPa) HEP (From 1.20 ± 0.43 to 4.20 ± 2.58 kPa) (liver tumor) (AFM)	[450–452]
Lung	5.6 (normal human lung) (a median pO2 value of 24 ± 6.4mmHg)	0.5–3 kPa (normal human lung) 100–1,000 Pa (Human lung tissue) (Ultrasound-based surface wave elastography)	5.6% (human lung tumor)	NA	[453–456]
Pancreas	7.5% (normal pancreas)	1 kPa (normal pancreas) (AFM)	0.3% (pancreatic tumor)	2 kPa (PanIN) and 4 kPa (PDAC) (AFM)	[267, 457]

In summary, hypoxia influences the mechanical properties and metastatic potentials of tumor cells and CAFsl. Additionally, although hypoxia is positively correlated with tumor bulk stiffness in many reports, one study shows the coexistence of central hypoxia and reduced local tissue stiffness in the center of early-stage breast tumors in the MMTV-PyMT mouse model [15]. It is conceivable that the central hypoxia in this study may be partially mediated by low local stiffness in the tumor core. Moreover, increased vasculature compression and decreased oxygen diffusion caused by solid stress may contribute to local hypoxia in the tumor core.

### Tumor cell mechanics

#### Mechanical alterations in primary tumor cells

Tumor cells show distinct mechanical properties, including Young’s modulus, viscoelasticity, membrane tension, contractility, and adhesiveness, in contrast to their healthy counterparts (Fig. 2). The changes in tumor cell mechanics are mainly contributed by a combination of altered cellular components, including the plasma membrane, cytoskeleton, nucleus, and the interactions among them.

#### Tumor cell stiffness

Accumulating evidence from clinical samples, xenografts, and cancer cell lines shows that the stiffness of cancer cells is generally lower than that of normal cells, with only a few exceptions (Fig. 2C). In the same patients, metastatic tumor cells of lung, breast, and pancreatic adenocarcinoma (0.53 ± 0.10 kPa) are 4-fold softer than normal cells (1.97 ± 0.70 kPa) [109]. Primary oral cancer cells are 3.5-fold softer than the cells from healthy donors [110]. Cancer cell lines, including bladder, breast, ovarian, lung, pancreas, prostate, and thyroid cancer, are 1.3—30-fold softer than normal cells [109, 111–115]. Nevertheless, metastatic pancreatic cancer and normal cells from patients share similar mechanical stiffness [109]. Only a few reports show that liver, prostate, lymphoid, and myeloid cancer cell lines are 1.4—2-fold stiffer than their normal counterparts [64, 116]. Interestingly, cervical cancer cells show both softening and hardening attributes with stiffness ranging from 2.5-fold softer to 1.4-fold stiffer than normal cells [117, 118]. It should be noted that all these stiffness measurements are conducted in vitro, which lacks the essential in vivo microenvironmental constitutes, and thus might not reflect their real mechanics within unperturbed tumors. One recent study combines particle tracking micro-rheology and intravital microscopy to measure tumor cell stiffness in vivo. The results show that the stiffness of cancer cells in the xenografts is higher than that on 2D substrates or in tumor spheroids encapsulated in 3D matrices [119]. Further, tumor cell stiffness is inversely correlated with metastatic and tumorigenic potential [120]. CSCs exhibit higher tumorigenic and metastatic potential but lower stiffness than nonCSCs in ovarian, skin, and breast cancer [37, 119, 121–123]. Breast cancer cell stiffness is inversely correlated with the invasiveness [70, 124, 125]. Patient-derived ovarian cancer cells with high invasiveness are 5-fold softer than the cells with low invasiveness [67]. However, highly invasive prostate cancer PC3 cells have higher Young’s modulus (1.4 kPa) than less invasive LNCaP cells (0.29 kPa), suggesting that the relations between cellular stiffness and malignancy may depend on cancer type [114].

Emerging findings show the dynamic changes of tumor cell mechanics throughout metastasis. Starting from tumor initiation, oncogenes can induce changes in the cytoskeleton of normal cells that lead to transient cell stiffening [22, 68, 69, 109, 110]. The mechanism underlying the switch from the transient stiffening in the oncogene-induced transformation to tumor cell softening in primary lesions remains unclear. Cancer cells that detach from the primary tumor and invade into the surrounding tissue become softer and larger at the invasive front in a tumor organoid model [70]. Notably, tumor cells and their nuclei become softened when migrating through constriction or endothelium [126]. After intravasation, circulating tumor cells (CTCs) adopt the cytoskeleton with lower fluidity to improve their lodging in the circulation [127]. The cancer cells surviving under shear flow show reduced stiffness and elongated morphology [128]. Of note, inhibiting actomyosin activity enhances the survival of CTCs in the vasculature. Upon the exit of the circulation system, increased stiffness of TRCs or CSCs lowers the extravasation rate [129]. The measurement from an isogenic panel of breast cancer cells shows that CTCs resemble parental tumor cells in their migration ability/cell stiffness, which is strikingly lower/higher than that of tumor cells metastasizing to the lung [130]. Further, the cytoskeleton and mechanics of breast cancer cells are correlated with their metastatic tropism [131]. However, to date no studies have systematically shown the alteration of tumor cell stiffness and their corresponding invasiveness throughout all stages of metastasis, mainly due to the lack of effective techniques with the capability to detect cellular stiffness in vivo. It is critical to elucidate whether these mechanical alterations at the cellular level provide causal and functional supports for tumor cells to adapt to distinct microenvironments at different stages and complete the entire metastatic journey.

#### Tumor cell contractility

Living cells generate contractile force via actomyosin machinery to actively probe and adapt to their microenvironment. Measurements of traction stress (in Pa), net traction force (in nN), strain energy (in pJ), the retraction characteristics of ablated cytoskeleton filaments, and matrix contraction of cells are instrumental in elucidating cellular contractility. The generation of cellular contractility depends on various intracellular and extracellular cues, including cell and ECM mechanical properties. Most reports show that compared to normal cells, cancer cells generate elevated traction force and exhibit enhanced motility, despite some exceptions (Fig. 2D) [132, 133]. For example, on soft substrates (2.83 kPa vs. 34.88 kPa), ovarian cancer cells show enhanced migration and proliferation with high traction force (620 Pa vs. 200Pa) [36]. In addition, cell spread area affects cell contractility. Cells with larger area generate larger force, but cell area alone does not dictate the magnitude of traction stress [134, 135].

Cellular contractility is associated with multiple cell functions, including invasion and migration. Tumor cells generate contractile force to invade the ECM or penetrate through the BM. Most reports show a positive correlation between contractility and invasiveness. For example, in the 3D condition, the contractile force at the rear part of breast cancer cells drives invasion [136]. Invasive breast and lung cancer cells have significantly higher contractility (2.9pJ and 0.8pJ, respectively) than noninvasive cancer cells (0.1pJ and 0.3 pJ) [137]. In the 2D condition, human metastatic breast, prostate, and lung cancer cell lines generate augmented net traction force (150–300 nN) compared to their nonmetastatic counterparts (80–160 nN) [134, 135]. Bladder cancer cells that generate higher traction force (170 Pa) exhibit increased invasiveness [138]. However, the opposite trend has also been reported. Our findings show that highly malignant colon cancer cells generate lower traction than less malignant cells [139–141]. Human lung cancer cells that are drug resistant show lower traction than control cells [142]. In mice breast cancer cell lines, increased metastatic potential is correlated with decreased traction (from 0.23 Pa to 0.05 Pa) [142, 143]. Our studies show that tumorigenic and metastatic TRCs and conventional tumor cells generate comparable magnitudes of traction. Further, high contractility suppresses tumor cell migration in several contexts. For example, elevating cellular contractility in glioblastoma stem cells enhances their mechano-sensitivity to substrate rigidity and suppresses local invasion in the brain [144]. Activation of myosin II increases cortical tension and contractility of pancreatic cancer cells and suppresses their migration [145]. One possible explanation to reconcile these contradictory correlations between invasiveness and contractility is that the relationship between migration velocity and traction force might not be monotonic [146]. The traction examined in different studies may fall into different segments of the relationship and lead to individually correct but mutually conflicting conclusions. Another explanation is that different levels of contractility are required to sustain the metastatic advantages of tumor cells at distinct steps during metastasis.

Contractility influences cell structure and signaling through mechanotransduction. Fibroblasts generate higher force at the perinuclear area than in the cell periphery due to the highly tensed actin-cap fibers. Such tensed fibers enable the nucleus to sense extracellular mechanical stimuli through the cytoskeleton, resulting in YAP nuclear translocation [147]. In another study, inhibition of myosin light chain kinase and contractility reduces YAP nuclear translocation, which can be rescued by additional stretching on the cell. However, once ROCK inhibition reduces contractility and eliminates the stretching-induced nuclear deformation, YAP translocation can no longer be rescued by stretching [148]. These findings suggest that contractility plays an instrumental role in maintaining cellular mechanical homeostasis and regulating cell behaviors [149].

#### Tumor cell adhesion

Cell adhesion molecules (CAMs) mediate cell-cell (e.g., Cadherin, selectin, and IgSF) and cell-ECM adhesion (e.g., integrin). During the metastasis process, tumor cells down-regulate epithelial markers (e.g., E-cadherin), up-regulate mesenchymal markers (e.g., N-cadherin), and lose cell-cell adhesion, which are related to EMT, a critical process in tumor metastasis [150–154]. Increased expression of E-cadherin is positively correlated with high cell-cell adhesion force and reduces the invasiveness of breast cancer cells [155]. P-cadherin affects the magnitude of intercellular adhesion force while E-cadherin regulates the rate at which the force builds up [156]. P-cadherin expression is correlated with cancer cell migration and invasion [157]. Integrins provide cellular anchors on ECM and is a driving force for cancer cells migration [158]. Cell-cell adhesion and cell-ECM adhesion are not often independent from each other. In human squamous carcinoma cells, disruption of the integrin-FAK-Src signaling suppresses the E-cadherin-regulated collective migration [159].

Both cell-cell and cell-ECM adhesion change dynamically during tumor progression. Reduced cell-cell adhesion often facilitates the detachment of tumor cells from the primary tumor during the early stage of metastasis (Fig. 2E) [146, 160, 161]. However, higher cell-cell adhesion is found in invasive breast cancer cells than normal cells [162]. This may be related to two different types of migration modes: single cell migration (reduced cell-cell adhesion) and collective migration (high cell-cell adhesion in the cell cluster). After the escape from the primary tumor, it is proposed that cell-cell adhesion is required in collective cell migration, the formation of CTC cluster, and the attachment of CTCs to endothelium for extravasation [163, 164]. On the other hand, transformed cells show lower adhesion to ECM than normal cells (Fig. 2F). For breast cancer cells, the subpopulation with high cell-ECM adhesion strength is less metastatic compared with the cells with low adhesion [165, 166]. Low adherent breast and colon cancer cells exhibit higher stemness in vitro and more tumor formation in vivo [167]. However, upon the arrival at the metastasized organ, the adhesions between CTC and BM and between CTC and endothelium are essential for successful generation of secondary tumors from disseminating cancer cells [168].

#### Cytoskeleton

Cytoskeleton primarily consists of actin filament, microtubule, and intermediate filament, and is considered a major contributor to the changes in cell stiffness, motility, and morphology [169, 170]. Different cytoskeletal elements bear different types of mechanical forces: F-actin and microtubule mainly bear tension and compression, respectively, while intermediate filament bears both. During tumor progression, cytoskeleton constantly remodels itself so that tumor cells acquire unique mechanical properties and can adapt to the dynamic changes in the encountered microenvironment.

Actin filaments show differential organization and polymerization in normal and cancer cells and are usually considered the major contributor to cell mechanics. Breast and ovarian cancer cells have looser actin organization and less stress fibers compared to normal cells, leading to the reduced tumor cell stiffness (Fig. 2G) [109, 112, 171, 172]. The formation of protrusions requires actin polymerization that is critical in cell migration and invasion [170, 173–175]. Invasive cells adopt an elongated spindle-like morphology with high density of actin filaments in the protrusion edge in 3D matrices [176]. The level of actin polymerization can be represented by the ratio of actin filament (F-actin) to globular actin (G-actin). Both high and low F/G actin ratios have been reported to be correlated to the invasiveness of colon cancer cells. Polymerization of microtubules leads to the increase of Young’s modulus in cancer cells and of shear modulus in pigment epithelial RPE1 cells [177–179]. ECM stiffening increases the stability of microtubules through glutamylation and facilitates breast cancer cells invasion [180]. The amount ratio of F-actin and microtubule and the interaction between them critically regulate cell mechanics and behavior. In migrating cells, actin provides the driving force at the protrusive cell front, while microtubules regulate the rear retraction [181]. Actomyosin contractility restricts the growth of microtubules in the protrusions at the early stage of fibroblast-ECM interaction. In colon cancer cells, higher F-actin/microtubule ratio correlates with higher malignant grade and cell stiffness.

Among the six types of intermediate filaments, vimentin is one important regulator of cancer cell migration and stiffness, while keratin and lamin also play important roles [182]. Oncogene-induced cell transformation leads to the increased width of vimentin fibers and the collapse of vimentin network, resulting in elevated cellular stiffness [183]. However, contradictory results about the effects of vimentin on cancer cell stiffness are reported. Ovarian cancer cells with higher vimentin appear to be softer [67]. Of note, increased expression of vimentin is found in stiffer breast cancer cells and facilitates EMT and cancer cell invasiveness [184–186]. Low expression of keratin is found during EMT with reduced cancer cell stiffness [187]. Lamin also regulates cell stiffness, and its effects will be further explained in the next section.

#### Nucleus

Nuclei often show irregular shape in cancer cells and ellipsoid shape with smooth contour in normal cells [188]. As the largest organelle in a cell, the nucleus often has 5–10-fold higher stiffness than the cytoplasm [189, 190]. Hence, nuclear deformability has an important role in tumor cell invasion. When disseminating tumor cells migrate through narrow constriction or dense TME, considerable levels of nuclear deformation are required and may lead to the transient rupture of nuclear envelope, potentially causing genomic instability, DNA damage, and cell death [191, 192]. For example, disseminating tumor cells that penetrate through the tissues with different stiffnesses may experience distinct amounts of nuclear deformation, which correlate with different levels of genomic instability and mutation in the tumors that originate from different tissues (Fig. 2H) [191–193].

Nuclear deformability largely arises from lamins within the nuclear envelope and chromatin [194, 195]. In single isolated nuclei, lamin A/C level and chromatin regulate nuclear strain stiffening at large (>3 μm) and small deformation (<3 μm), respectively. The ratio of euchromatin/heterochromatin modulates nuclear stiffness [196]. Distinct nuclear mechanics may be required at different stages of tumor progression. During tumor initiation, knockdown of lamin A/C in human neuroblastoma cells increases the population of tumor-initiating cells [197]. The occurrence of nuclear rupture is more frequent in lamin A/C-deficient mouse embryonic fibroblasts compared to that in wild-type control [198]. Lamin-regulated nuclear stiffness is proposed to protect normal cells from force-induced nuclear rupture and mutation, which may prevent tumor initiation. However, this mechanism might be independent of the effect of lamins on nuclear mechanics. For example, lamin A enhances DNA repair by regulating p53-351 binding protein-1 (53BP1) [199]. Knockdown of lamin-A enhances the nuclear softness by 4-fold and promotes tumor growth in the lung tumor xenografts [200]. During migration, intravasation, and extravasation, tumor cells need to squeeze through the confinement with softened nuclei. When transiting through the circulatory system, lamin A/C protects CTCs from the shear-induced destruction in the vasculature [201]. However, the role of nuclear mechanics in metastatic colonization remains a mystery.

#### Cell membrane

Besides the dominant roles of nucleus and cytoskeleton, cell membrane also contributes to cell stiffness [170]. Cell membrane stiffness is influenced by membrane bending rigidity, fluidity, membrane tension, and the connection with the underlying cortex [202]. It is usually quantified by measuring the shape fluctuation of the membrane: higher fluctuation represents lower stiffness. Regulated by the membrane lipid composition and gradients of sphingolipids and cholesterol, cancer cells often show lower membrane stiffness than normal cells that inversely correlates with invasiveness. For example, primary breast and cervical tumor cells show softer cell membranes compared to their normal counterparts (Fig. 2I) [203]. The invasiveness of breast cancer cells decreases when the cell membrane stiffness increases [204]. In highly metastatic T-lymphoma cells, cell membrane fluctuation is higher than its corresponding nonmetastatic cell lines [205]. Cell membrane fluidity (viscosity) is a mechanical property that describes the time-dependent molecular motion and level of molecular disorder within the membrane [206]. The fluidity of cell membrane correlates with membrane tension, cell proliferative potential, and poor prognosis in liver and lung cancer [207, 208]. In breast cancer cells, EMT-mediated membrane fluidization can increase cancer cell motility [209]. The mechanics and function of cell membrane highly depend on the underlying cytoskeleton [210]. In cancer cells, the membrane-to-cortex attachment maintains the membrane tension and inhibits the migration and invasion through the curvature-sensing BAR proteins [137, 210]. Actin-polymerization-induced protrusion causes a temporary increase of membrane tension and unfolding of the membrane wrinkling [211]. Cell membrane stiffness and the function of membrane proteins can reciprocally affect the cortex cytoskeleton structure. During migration of neutrophils, membrane tension at the leading-edge increases, inhibiting the formation of the secondary fronts by actin assembly. Therefore, the mechanical properties and function of cell membrane need to be investigated together with the cytoskeleton.

### Summary

The mechanics of tumor tissues and tumor cells considerably change during tumor progression. These multi-scale mechanical alterations regulate gene transcription and protein activities, as well as influence tumor cell functions and malignancy through mechanotransduction. However, several fundamental questions remain to be addressed.

First, tumor cells are generally softer than healthy cells, while tumor tissues are stiffer than healthy tissues. However, what is the sequential process of these mechanical alterations during tumor initiation and progression? How can the alterations in tumor cell mechanics contribute to the mechanical changes in tumor tissues, or vice versa?

Second, the dynamic alterations of tumor cell stiffness during metastasis are still not comprehensively understood. The underlying driving force remains elusive. It is possible that tumor cells actively alter their mechanical properties to adapt to different microenvironments during metastasis. Alternatively, various factors in the tumor microenvironment, including mechanical cues, likely select a subpopulation of tumor cells that have unique cellular stiffness and metastatic advantages. The functional roles of cellular stiffness at each stage of metastasis remain unknown. It is worth investigating whether the mechanics of tumor cells function synergistically or independently with the biophysical contexts of tumor tissues. The therapeutic role of tumor cell mechanics in mechanotargeting is yet to be explored.

Third, the mutation rate of a tumor is correlated with tissue mechanics, and high matrix rigidity is required for the oncogene-induced cell transformation. However, how the dynamic alterations of tissue mechanics influence gene mutation and facilitate tumor initiation remains unclear. The role of tumor cell stiffness in the tissue mechanics-mediated gene mutation remains unknown.

Fourth, disseminating tumor cells metastasize to specific organs, i.e., organotropism. One hallmark of metastasis is the establishment of a favorable premetastatic niche for hosting the disseminating tumor cells. However, it remains unclear whether and how primary tumor cells with specific organotropism influence the mechanics of the target organs before their arrival. Considering the mechanical heterogeneity of the primary tumor, we hypothesize that mechanically heterogeneous local niches in the primary tumor possibly confer resident tumor cells the ability to metastasize to different organs. These mechanically primed tumor cell subpopulations may adjust the mechanics of the metastasized organs and establish a favorable premetastatic niche to support the survival and outgrowth of the arriving tumor cells, which contributing to metastatic tropism.

## The crosstalk between mechanotransduction and oncogenic signaling

### Mechanotransduction

Cells not only experience various types of mechanical cues but also actively exert endogenous forces on their surroundings [212]. Subjected to the mechanical stimuli, intra- and extracellular biomolecules change their conformations and alter the downstream biochemical signaling. The mechanisms by which cells sense and transduce mechanical cues into biochemical signals and events are referred to as mechanotransduction. For example, recent data suggests that substrate stiffness can regulate the dynamics of intra/intracellular calcium signals [213]. On one hand, these two types of signaling share many common intracellular pathways and can work in parallel for the same function, such as force-induced Rac activation that is independent of canonical Src activity [214]. On the other hand, different from chemo-transduction, mechanotransduction has its own characteristics, including distant force propagation, directional specificity, and the rapid rate of force generation, transmission, and halt.

#### Mechanotransduction in cancer

Mechanotransduction depends on cell mechanics and force transmission machinery. As summarized in Section “Mechanics in tumor growth,” tumor cells show considerable alterations of physical properties, which underline their unique mechano-sensitivity. The stiffness/spreading/proliferation/metabolism of normal cells increase as their surrounding ECM stiffens, while those of tumor cells often respond less potently. For example, Ha-RasV12-transformed cells maintain constant cellular stiffness and proliferation on the substrates with varying rigidities. nonsmall-cell lung cancer cells show a consistently high glycolytic rate regardless of the microenvironmental stiffness, while human bronchial epithelial cells increase glycolytic rate on the substrates with increasing rigidity [215]. Noticeably, the distinct mechano-response of cancer cells appears to depend on the dimensionality of the environment. For example, MDA-MB-231 cells show rigidity-dependent growth on 2D substrates, while displaying similar morphology and growth rate in 3D environments of varying rigidities. Moreover, most CSCs exhibit even lower mechano-sensitivity compared to nonCSCs. Glioma tumor-initiating cells display unaltered expressions of stemness markers, spread area, and proliferation on the substrates with different stiffnesses compared to conventional cancer cells. Fibrin-selected TRCs, but not unselected cancer cells, maintain their stiffness unchanged on the substrates of different stiffnesses due to the reduced level of Cdc42 [38]. In addition, cancerous and healthy cells respond differently to other types of mechanical cues. For example, normal fibroblasts, but not fibrosarcoma cells, reverse the migration direction within the microchannels in response to fluid shear stress through TRPM7 and RhoA activities. Confinement-induced chronic DNA damage causes cellular senescence of normal cells, but promotes invasion of transformed cells [193]. This divergence might be because normal cells have stiffer nuclei and higher expression of Lamin A/C than tumor cells.

To date, at least three distinct mechanisms have been discovered underlying the unique mechano-sensitivity of tumor cells. First, Ha-RasV12–transformed pancreatic, breast, and kidney cells alter their mechano-sensitivity to substrate stiffness due to the absence of Caveolin-1 (Cav1) [117]. Second, breast and many other cancer cells lose tropomyosin 2.1, a protein involved in stabilizing actin filaments and binding proteins, and show diminished rigidity sensing. Third, glioma tumor-initiating cells lack the mechano-sensitivity to ECM stiffness because of insufficient myosin-dependent contractility. Of note, the mechano-sensitivity of tumor cells to substrate rigidity can be restored by re-expressing Cav1 or tropomyosin 2.1 or increasing cell contractility, indicating the dominant role of each mechanism.

Interestingly, some tumor cells appear to be more sensitive to certain mechanical cues. For example, cyclic stretching increases the apoptosis of rigidity-independent transformed cells through Piezo1-mediated intracellular Ca^2+^ overloading but elevates the proliferation of normal cells [216]. Low-frequency ultrasound (33 kHz) suppresses the growth and induces apoptosis of tumor cells by up-regulating Piezo1 but has minimal effects on normal cells. However, why the intracellular Ca2+ signal in transformed cells is hyper-sensitive to cyclic stretch and ultrasound requires further investigation.

#### Mechanical cell competition in cancer

Normal epithelial cells often compete with transformed cells for survival, which can be affected by mechanical stress and cell compaction through either eliminating or expanding the transformed cells.

At the early stage of cancer, when normal cells surround transformed cells, they form arm-like vimentin and generate contractile force to prevent the enclosed transformed cells from forming basal extensions and extrude the transformed cells out from the top of the epithelial monolayer. Wild-type cells can aggressively sequester, compact, and eliminate Scribble-deficient cells through the elevated p53 only when being surrounded by but not in simple contact with wild-type cells, suggesting that mechanical but not only biochemical cues within the contact interface drive this process [217]. Consequently, all the transformed cells should arguably be eradicated so that no overt tumors can be generated. However, this argument does not agree with the prevalence of cancer, indicating that other factors are present to counteract the mechanical extrusion during tumor initiation, including ECM rigidity and cyclic strain [218]. For example, rigid ECM (90 kPa) inhibits the extrusion of HRasV12-transformed cells from the epithelial monolayer compared to soft ECM (4 kPa) via filamin re-localization [218]. Cyclic strain (1 Hz, 3–9% strain amplitude) prevents the apical extrusion of transformed cells from the healthy monolayer while facilitating their basal invasion due to the diminishment of the functional difference in cortical actin between RASV12 transformed cells and wild-type cells [219].

Tumor cells can not only evade mechanical cell competition but also eliminate healthy cells. During the larval competition in Drosophila imaginal wing disks, cells with elevated proto-oncogene dmyc (winner) outcompete wild-type cells (loser) by extruding them from the epithelium due to different growth rate and high tension in the interface. Different tension is caused by variations in F-actin level at the connective points between losers and winners. Moreover, in Drosophila pupal notum, RasV12-transformed clones cause ectopic tissue compaction of neighboring wild-type cells, triggering their extrusion and death by down-regulating ERK signaling. Release of the tissue stress reduces cell compaction and transiently activates ERK signaling, thereby preventing the extrusion of wild-type cells [220]. Further, overexpressing the active form of MyoII but not blocking the paracrine factors of the RasV12 clone reduces wild-type cell elimination, suggesting that cell elimination is driven by mechanical rather than chemical cues [220]. Nevertheless, how the physical alternations during tumor initiation and progression influence the interaction force between normal and tumor cells remains incompletely understood.

Cancer cells compete mechanically with normal cells, which crucially influences tumor progression. Abnormal alterations in the mechanics of TME can facilitate cancer cells to outcompete normal cells [221–224]. However, the underlying mechanisms remains poorly understood. Hence, understanding the mechanotransduction mechanism underlying the influence of tumor-specific mechanical environments on the cell competition is crucial. Recent studies show that mechano-triggered intercellular calcium waves facilitate the extrusion of oncogenic transformed and apoptotic cells through the reinforced actin ring structures [221]. Targeting the mechanotransduction may help suppress tumor development from a new perspective.

### Mechanosensitive proteins and their crosstalk with oncogenic signaling in tumor growth

**Fig. 3:**
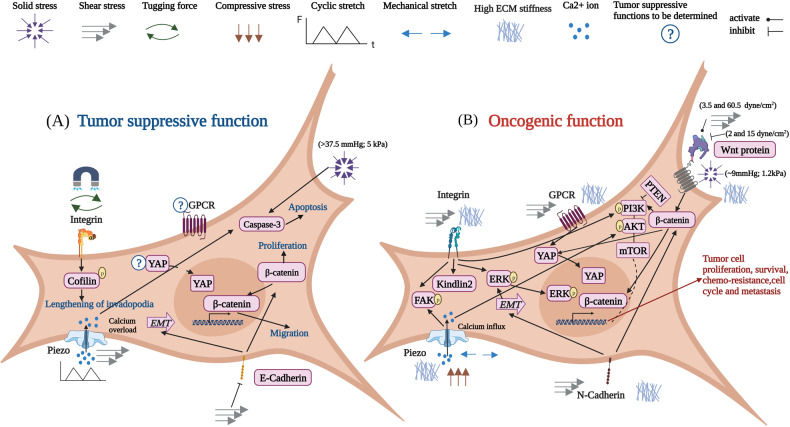
Mechanosensors and mechanosensitive proteins exhibit oncogenic and/or tumor suppressive effects on tumor progression. **A** 1-Integrin: The stiffened matrix activates integrins and their downstream FAK, PI3K and ERK signaling, which promote tumor cell proliferation, growth, and metastasis [230, 437, 438]. Fluid shear stress suppresses integrin β1-FAK signaling to enhance the radiation-induced cell death [201]. 2-Piezo channels: Compressive stress and mechanical stretch activate Piezo1 to induce calcium influx, which triggers the downstream FAK and AKT signaling to enhance tumor cell proliferation and invasion [244, 245]. 3-N-Cadherin: High matrix stiffness and fluid shear stress upregulate N-cadherin to induce EMT and facilitate tumor cell migration [239, 241]. 4-Wnt/β-catenin: Tumor growth-induced solid stress (~1.2 kPa;) activates Wnt/β-catenin pathway in healthy mouse colon tissue, which leads to hyperproliferation and formation of aberrant foci [439]. Elevated ECM stiffness activates Wnt/β-catenin pathway and downstream PI3K and YAP signaling to facilitate tumor cell growth and chemoresistance [29, 267]. Increased shear stress from 3.5 dyne/cm^2^ to 60.5 dyne/cm^2^ activates β-catenin to influence cancer cell proliferation [440]. 2 dyne/cm^2^ and 15 dyne/cm^2^ shear stress suppresses Wnt/β-catenin signaling to induce differentiation of CSC or cell cycle arrest [441, 442]. 5-GPCR: Breast cancer cells respond to shear stress through GPCR [250]. High matrix stiffness facilitates HCC cell proliferation and stemness through GPCR-induced YAP activation [253]. **B** 1-Integrin: Tugging force that is applied through magnetically actuated matrix inhibits integrin activity and increases cofilin activity to induce lengthening of invadopodia [228]. 2-Piezo channels: Cyclic stress and shear stress activate Piezo and trigger the overloading of calcium to induce tumor cell apoptosis [216]. 3-E-Cadherin: High matrix stiffness and shear stress down-regulate E-cadherin to induce EMT or through β-catenin pathway to facilitate tumor cell proliferation and migration [237, 239, 267]. 4-High solid stress (over 37.5 mmHg) suppresses cancer cell proliferation and induces cell apoptosis [86–88].

Mechanosensors refer to the biomolecules, such as integrin, cadherin, Piezos, and GPCR, that utilize their force-induced conformational changes or modifications to convert mechanical cues to biochemical signaling. Mechanosensitive biomolecules are the molecules that can respond to physical stimulations but have not been verified as mechanosensors by any direct evidence, such as YAP/TAZ and EMT-associated proteins. The oncogenic (Fig. 3A) and tumor suppressive (Fig. 3B) functions of mechanosensors and mechanosensitive proteins are summarized in Fig. 3. As described in “Mechanics in tumor growth,” the mechanics of TME dynamically change during tumor progression. Thus, how the mechanosensors and mechanosensitive biomolecules in tumor cells sense and respond to the altered physical stimulations critically influences tumor cell behaviors and functions. These mechanotransduction activities interact with classical oncogenic signaling, synergistically affecting tumor progression. Conversely, the altered mechanics of TME and tumor cell mechanotransduction can reinforce the mechanosensing machinery.

#### Integrin

As heterodimeric transmembrane receptors, integrins have α and β subunits that bind to specific ECM ligands. Upon the binding of the α subunit with ECM, the β subunit interacts with cytoskeleton in the intracellular domain to activate the downstream signaling [225]. Many clinical findings have shown a close correlation between high expression of integrins and poor survival of cancer patients, despite a few exceptions as detailed in Table 3.

**Table 3:** Functions of different subtypes of integrin in mechanotransduction.

Integrin subtype	Effects on tumor progression	Cell type	Alterations in tumor tissue and tumor cell mechanics	Mechanism	Refs
β1	Increase tumor latency and decrease tumor incidence	Ha-ras MCF10 cells	Decrease tissue stiffness	PI3K signaling	[53]
β1	Promote cancer cell proliferation	Colorectal cancer cell	Increase matrix stiffness	Increase FAK/SRC phosphorylation	[437]
β1	Increase tumor cell motility	GBM cells	Increase substrate stiffness	Glycocalyx bulkiness; FAK	[230]
β1	Decrease lung metastases	CAF	Decrease tumor tissue stiffness	DDR2	[232]
β1	Promote migration	Breast cancer cells	Increase substrate stiffness	β1-FAK activation mediated RhoA/ROCK1/p-MLC and RhoA/ROCK2/p-cofilin pathways	[458]
αvβ3	Increase the invasiveness of cancer cells in 3D	Breast, melanoma, Kidney and bladder carcinoma cells	Increased cellular stiffness	NA	[459]
αvβ3	Promote tumor invasion	CAF	ECM remodeling	Increased fibronectin assembly	[460]
α5β1	Promote malignant phenotype	Mammary epithelial cells	Stiffer ECM	PI3K/ERK pathway activation	[438]
α5β1	Promote invasion	Breast cancer cells	Increased cellular stiffness and contractile forces	Increased membrane-type 1 MMP	[461]
α9β1	Promote migration and lung metastasis	Human GBM and osteosarcoma cells	ECM glycoprotein tenascin C	Inhibit actin stress fiber formation and YAP expression	[462]
β3	Induce the dormancy of tumor-repopulating cells	Melanoma cells	3D matrix stiffening	Facilitate Cdc42 into nucleus and promote Tet2 to activate p27 and p21	[463]
β3	Promote invasion	Fibrosarcoma cells	Transient tugging forces	Higher activity of cofilin, elongated invadopodia	[228]
α3β1	Awaken dormant cancer cells	Breast and prostate cancer	Laminin remodeling	Sustained inflammation induced the formation of neutrophil extracellular traps	[464]
α6β4	Promote malignant phenotype	Mammary epithelial cells	Stiffer ECM	PI3K/Rac activation	[48]
α2β1	Facilitate invadopodia formation	Breast cancer cells	Dense 3D collagen	NA	[186]
α11	Inhibit tumor growth and metastatic potential	CAF	Softer tumor tissue	NA	[465]

Integrins are critical mechanosensors in tumor cells that perceive the mechanical alterations in the TME [226]. Breast myoepithelial cells generate a peak contractile force on 1 kPa fibronectin-coated substrates via both α5β1 and αvβ6 integrins when matrix stiffness increases from 0.1 kPa to 10 kPa. Integrin-β6-overexpressing myoepithelial cells that often exist in ductal carcinoma generate the highest contractility on 1 kPa only via α_5_β_1_ integrins, 5 kPa only via α_v_β_6_ integrins, and 10 kPa via both integrins, respectively, suggesting that different integrin sub-types determine distinct rigidity sensing [227]. Tugging forces exerted via magnetic beads decrease β3 expression and increase cofilin activity to promote the invasion of fibrosarcoma cells (Fig. 3B) [228]. The stiffened tumor tissue activates integrin and downstream signaling, which often confers tumor cells to a more malignant phenotype [11, 229]. Inhibiting lysyl oxidase (LOX)-mediated collagen crosslinking decreases tissue fibrosis and stiffness to reduce tumor incidence via β1 integrin-PI3K signaling [53]. Elevated tissue stiffness leads to a bulky glycocalyx on the cell membrane that regulates the integrin clustering and integrin-ECM interaction to activate FAK and ERK pathways, which enhance the survival and growth of breast and brain tumor cells (Fig. 3A) [230, 231]. The integrin-mediated mechanotransduction reciprocally influences the mechanics of TME. Collagen cross-linking is associated with stromal integrin α11 expression. In nonsmall cell lung carcinoma (NSCLC) cells, loss of integrin α11 correlates with decreased collagen reorganization and tissue stiffness, which inhibits the growth and metastatic potential of NSCLC cells [200]. Depletion of DDR2 in breast CAFs alters organization of collagen fiber and decreases both tumor tissue stiffness and β1 integrin activity, leading to reduced lung metastases [232].

In addition, integrins can respond to other types of mechanical cues. For example, fluid shear flow induces G2/M arrest in osteosarcoma cells by activating αvβ3 and β1 integrins [233]. In colon cancer cells, shear stress suppresses integrin β1-FAK signaling and enhances the radiation-induced cytotoxicity (Fig. 3A) [201]. In breast cancer cells, low shear stress facilitates internalization of integrin β1 to promote cell migration [197]. During metastasis, different integrin subtypes may have different roles. We have summarized the different mechanotransduction functions of integrin subtypes in Fig. 3 and Table 3. Even the same subtype of integrin may have both oncogenic and tumor suppressive effects on biological and mechanotransduction signaling (Table 4). Their contradictory activities add another layer of complexity and challenge into the integrin-targeting cancer therapy, which may partially explain the failure of targeting single specific integrin in clinical trials. Further investigation of integrin-mediated mechanotransduction in tumor progression is needed to elucidate the comprehensive functions.

**Table 4:** Distinct tumor-suppressive and oncogenic functions of integrins.

Integrin subtype	Biological functions	Mechanotransduction	Refs
α2β1	Loss of α2β1 integrin promotes breast cancer metastasis in vivo and α2β1 integrin over-expression inhibits migration, intravasation, and anchorage-independent growth in vitro.	High-density fibrillar collagen (HDFC) matrix promotes invadopodia in breast fibrosarcoma and prostate carcinoma cell lines and in primary human fibroblasts by activating α2β1.	[229]
β3	Melanoma tumor growth and angiogenesis are enhanced in β3 deficient mice.	Tugging forces using magnetic beads in vitro decrease β3 expression and promote invasion of fibrosarcoma cells.	[228]
β3	The β3+ -richsubpopulation cells from patient-derived lung and pancreatic xenografts show tumor-initiating cell properties and chemoresistant ability through KRAS/RalB/NFκB pathway.	Increased 2D matrix stiffness elevates β3 expression of breast cancer cells and tumor-produced factors that are associated with bone destruction (Gli2 and PTHrP).	[466]
β1	Inhibiting integrin β1 expression in lung cancer cells show decreased lung tumor number and volume in mice through c-Met/RTK pathway.	Increased matrix stiffness facilitates β1 integrin clustering and promotes focal adhesions to drive invasion of Ha-ras mammary epithelium in vitro and in vivo.	[53]

#### Cadherin

Cadherins (E-, N-, VE-, P-, R-, and K-cadherin) are transmembrane proteins that function as cell-cell interaction receptors and mediate calcium-dependent adhesion. Downregulation of E-cadherin is associated with tumor initiation and progression, whereas high N-cadherin expression promotes malignancy in a variety of tumor types [199]. The roles of R-/K-cadherin in cancer are under active investigation.

As a mediator of cell-cell interaction, cadherins are important mechanosensors that perceive and transmit mechanical cues generated by adjacent cells. The cadherin cytodomain connects with actin cytoskeleton through β-catenin and α-catenin to mediate mechanotransduction [234]. E-cadherin–mediated force transduction impacts various cellular functions. Force transmission via E-cadherin activates epidermal growth factor receptor (EGFR) signaling, which is responsible for local cytoskeletal remodeling and cell proliferation [235]. In MCF-10A cells, tension on the E-cadherin bond disrupts the EGFR/E-cadherin complex on the cell membrane in the absence of epidermal growth factor (EGF) [236]. EGFR monomers released from the complex facilitate the binding of EGFR with EGF to activate the downstream signaling [236]. Further, stiff 3D microenvironments (310 Pa vs. 30 Pa) reduce E-cadherin expression, disrupt the colocalization of E-cadherin with β-catenin, and promote the proliferation and invasion of lung and gastric tumor cells (Fig. 3B) [237]. However, 3D stiff collagen-PEG gels (4 kPa vs 0.7 kPa) instruct the formation of tumor spheroids of hepatocellular carcinoma cells and reduce tumor cell malignancy while enhancing E-cadherin expression localized at the cell-cell boundaries [238]. These contradictory findings are possibly due to the difference between the absolute values of the matrix stiffness and different cancer types. Moreover, shear force enhances N-cadherin expression and decreases E-cadherin expression that can release β-catenin from E-cadherin and translocate it into the nucleus, thereby promoting the migration and invasion of breast, liver, and oesophageal cancer cells (Fig. 3A, B) [239, 240]. Stiff ECM up-regulates N-cadherin on the surface of endothelial cells, which enhances the interaction between tumor cells and vascular endothelium to promote metastasis (Fig. 3A) [241].

In summary, cadherin-mediated cell-cell interaction is essential for the migration, survival, and growth of cancer cells. However, how cadherin-mediated mechanotransduction affects tumor progression in vivo remains to be investigated.

#### Piezos and other ion channels

Piezo1/2 ion channels (Piezo1 and Piezo2) are mechanosensitive transmembrane proteins conserved across eukaryotes. Specifically, under membrane tension (~92 nN), Piezo1/2 channels undergo conformational changes from curved to flatten states, followed by the extracellular calcium influx and intracellular calcium release to influence cellular functions [242]. The aberrant expressions of Piezo1/2 are either positively or negatively correlated with patient survival in a variety of cancer types, implicating that Piezo1/2 show either oncogenic or tumor suppressive functions, likely depending on cancer type and subtype [243].

As mechanosensors, Piezo1/2 perceive external mechanical cues to influence the proliferation, migration, apoptosis, and invasion of cancer cells. For example, mechanical stretch activates Piezo1 and its downstream Akt/mTOR pathway, which promotes cell cycle progression in prostate cancer cells. Glioma tumor tissue stiffening activates Piezo1 at focal adhesions, which further facilitates integrin-FAK signaling, remodels ECM, and reinforces tissue stiffening (Fig. 3A) [244]. In breast cancer cells, mechanical compression activates Piezo1 and RhoA/Src/FAK/ERK signaling, which subsequently enhance tumor cell invasion, matrix degradation, and invadopodia formation (Fig. 3A) [245]. ECM stiffness activates Piezo2-mediated calcium influx in breast cancer cells preferentially metastasizing to the brain, which maintains the activation of RhoA, the orientation of stress fibers, and focal adhesions [246]. Force-induced calcium influx via Piezo1 can be exploited to target cancer cells [216]. For example, circulatory shear stress promotes Piezo1-mediated calcium influx, which sensitizes suspended prostate, breast, and colon cancer cells to TRAIL-induced apoptosis (Fig. 3B) [216]. Together, the roles of Piezo1/2 as mechanosensors are crucial in tumor progression. Their comprehensive functions in different types of cancer during different tumor stages are to be elucidated.

#### G protein-coupled receptors (GPCRs)

As transmembrane proteins, GPCRs have seven transmembrane α helix structures that couple to heterotrimeric G-protein (Gα, Gβ, and Gγ subunits). Following ligand-receptor activation, cytoplasmic GPCR kinase phosphorylates the corresponding receptor, such as dopamine and GABA receptors, and triggers G-protein-independent GPCR signaling cascades [247]. Clinical findings indicate the positive association between high expressions of GPCRs and poor patient survival, but also show the tumor suppressive functions of GPCRs in glioblastoma, breast cancer, and endometrial cancer [248].

GPCRs function as mechanosensors with the ability to sense and respond to mechanical forces. As an endothelial mechanosensor, the structure Helix 8 is required to sense fluid shear stress [249]. Recently, the role of GPCRs as mechanosensors in cancer cells has been increasingly uncovered. For example, breast cancer cells sense 2-Pa shear stress via GPR68/OGR1 to trigger calcium transients, while 24-Pa pressure inactivates GPCRs to regulate F-actin assembly and YAP activity (Fig. 3A) [250, 251]. Breast cancer cells on 350-kPa hydrogels exhibit a higher unbinding force of a GPCR family member CXCR4 than normal mammary cells [252]. When matrix stiffness increases from 1 kPa to 6 kPa and 12 kPa, HCC cells upregulate CXCR4 and decrease UNTD1 activity to facilitate proliferation, EMT phenotype, and CSC features (Fig. 3A) [253]. Interstitial flow at 0.7 µm/s and 0.1 µm/s increases cell invasion in glioblastoma and hepatocellular carcinoma through activating CXCR4 [254, 255]. GPCRs also influence tumor tissue mechanics to affect tumor progression. For example, GPR56 binds to TG2 to inhibit its ECM crosslinking function and to remodel fibronectin, leading to the inhibition of melanoma growth. Tamoxifen, an agonist of the GPER, decreases tumor stiffness through GPER-RhoA-YAP and MLC2 signaling. GPERs-mediated ECM remodeling deactivates pancreatic stellate cells and inhibits tumor cell invasion [256]. Further studies of GPCRs as mechanosensors in tumor progression are needed to reconcile the reported contradictory findings. For example, high expression of GPR56 is associated with poor prognosis in CRC but with favorable patient survival in glioblastoma [257, 258]. Since the stiffness of colon and brain tissues varies, investigations are required to clarify whether GPR56 or other GPCR proteins function distinctly under different mechanical stimuli and contribute to tumor progression via different mechanisms.

#### Epithelial–mesenchymal-transition-associated signaling

Epithelial–mesenchymal transition (EMT) represents the transition of polarized epithelial cells towards the mesenchymal state, which involves reduced expression of epithelial markers (E-cadherin and cytokeratin) and enhanced expression of mesenchymal markers (N-cadherin, Twist, Vimentin, Snail, and Slug) [259]. On one hand, EMT promotes metastatic potential and confers CSCs properties on transformed cells [260]. EMT is positively/negatively associated with tumor grade/patient survival [261]. On the other hand, deletion of Snail1 or Twist1 in mice does not influence metastasis but increases the sensitivity to chemotherapy, which indicates that EMT is possibly indispensable in drug resistance but not metastasis [262]

The EMT phenotype of tumor cells is sensitive to not only biochemical but also mechanical cues [259]. In breast cancer, elevated substrate rigidity (from 150 Pa to 5700 Pa) facilitates EMT and cancer cell invasion via TWIST1–G3BP2 and EPHA2/LYN/TWIST1 pathways [263]. High stiffness (~16 kPa vs. ~9 kPa and ~5 kPa) of liver tissue in the rat model induces EMT through eIF4E in HCC [264]. OSCC cells with high E-cadherin/N-cadherin ratio gain mesenchymal characteristics after 5-day culture in stiff (20 kPa vs. 0.48 kPa) microenvironments and exhibit enhanced migration [265, 266]. In colorectal cancer cells, elevated substrate stiffness (2, 10, 40, and 95 kPa) facilitates the secretion of TGF-β member Activin A and induces EMT to promote migration [266]. In pancreatic cancer cells, stiff substrates (25 kPa vs. 4 kPa) upregulate Vimentin, downregulate E-cadherin, and enhance YAP/TAZ nuclear localization, which may enhance tumor cell resistance to paclitaxel (Fig. 3B) [267]. Interestingly, ovarian cancer cells undergo EMT with high motility on soft substrates (2.83 vs. 34.88 kPa) [36]. Further, fluid shear stress facilitates the EMT phenotype of LSCC, breast cancer, ovarian cancer, and enhances various cellular functions, including migration, motility, stemness and tumor formation, and the survival in blood circulation [83, 268]. In renal cell carcinoma, accumulated solid stress (4 mmHg) facilitates EMT through Akt-GSK-3β-β-catenin pathway [83].

EMT remodels cell cytoskeleton and potentially influences tumor cell stiffness and contractility. In 3D microenvironments, the stiffness of head and neck cancer cells is inversely correlated with EMT phenotype and invasiveness [269]. nonsmall cell lung cancer cells treated with Epigallocatechin gallate exhibit reduced motility and Vimentin/Slug and increased cell stiffness [270]. TNF‐α‐inducing-protein (Tipα) promotes gastric cancer cell motility and increases Vimentin expression with decreased cellular stiffness [271]. On the contrary, over-expression of Vimentin in breast cancer cells induces EMT and increases cell stiffness [184]. These findings suggest that the influence of EMT on cell mechanics is likely cancer-type-specific. Interestingly, the induction of EMT decreases/increases the stiffness and contractility of breast cancer cells in interphase/mitosis [272].

In summary, mechanical stimuli influence the EMT phenotype and metastatic potential of tumor cells [239, 263]. Systematic investigations on the causal effects of different magnitudes and types (e.g., compression, shear, and tension) of mechanical cues on EMT can further elucidate the role of mechanics-induced EMT in metastasis.

#### YAP/TAZ-mediated mechanotransduction signaling

YAP (Yes-associated protein) and TAZ (transcriptional co-activator with PDZ-binding motif) are orchestrated by the Hippo signaling [273] and show both oncogenic and tumor-suppressive effects. On one hand, high mRNA and protein levels of YAP/TAZ associate with poor prognosis of patients in nonsmall-cell lung cancer, HCC, melanoma, glioma, colon cancer, and breast cancer [274]. On the other hand, YAP is down-regulated in hematological and breast cancer cells, and its low expression correlates with poor patient survival [275, 276]. One recent study shows that overexpression of YAP can promote and suppress tumor growth in the YAP-expression and YAP-lacking tumor models, respectively [277]. These opposing findings suggest the instrumental and multi-faceted roles of YAP/TAZ in cancer. Importantly, YAP/TAZ respond to not only biochemical but also mechanical stimuli, including stiffness/topology of ECM, shear/compressive stress, cytoskeletal prestress, and cell density.

ECM stiffness regulates YAP activation in various tumor cells mainly through cytoskeletal tension. On one hand, stiff gels (3 vs. 1.2 mg/ml) inhibit F-actin-capping/severing proteins to promote cytoskeleton tension and translocate YAP/TAZ into the nuclei of transformed MCF-10A cells, which is independent of Hippo signaling [278]. ECM stiffness facilitates YAP nuclear localization through CXCR4/UBTD1 signaling in HCC (Fig. 3A) [279]. In addition, matrix stiffness reversibly regulates DNA methylation in the promoter region of YAP in gastric cancer cells [280]. The rigidity-dependent YAP response has been exploited to specifically kill breast cancer cells at the stiff lung metastatic sites [281]. Meanwhile, YAP activation of cancer cells on stiff ECMs is often associated with increased spread area and focal adhesion (FA) [282, 283]. Altering adhesion area does not affect YAP nuclear localization when cell spreading is constant, which indicates that the effect of substrate rigidity on YAP activity is not through FA. Whether this effect is mediated by cell spreading is still to be determined. On the other hand, soft substrates activate RAP2 and LATS1/2 that retain YAP/TAZ in the cytoplasm of breast cancer cells, inhibiting the formation of aberrant acini, anchorage-independent growth, and xenograft growth [284]. The reduced YAP activity in HCC cells on soft ECMs can be rescued by proteoglycan Argin through inhibiting Merlin, LATS1/2, and ILK-PAK [285]. These findings indicate that soft and stiff ECMs induce YAP cytoplasmic and nuclear translocation are dependent on and independent of Hippo pathway, respectively. Further, 3D soft fibrin gels retain YAP in the cytoplasm of tumor cells, promoting their growth and stemness [286, 287]. Interestingly, several recent reports show that increased stiffness of 3D matrices enhances the malignancy of breast cancer cells but does not affect YAP nuclear translocation [288]. Subcellular localization of YAP in fibroblasts is independent of substrate stiffness, but determined by nuclear deformation [56]. Further, 3D rigid collagen/alginate hydrogels (16.85kPa) down-regulate YAP1 and retain YAP in the cytoplasm in breast cancer cells compared to soft hydrogels (2.27 and 3.94 kPa) [289]. These contradictory findings may be due to the different ranges of matrix stiffness and the specific gel properties.

YAP/TAZ activity is also sensitive to other mechanical cues. For example, fluid shear stress promotes YAP nuclear localization to enhance the proliferation and motility of prostate cancer cells. In HCC cells, fluid shear stress facilitates cytoskeleton rearrangement that releases YAP from integrin β1, initiates YAP nuclear translocation, and promotes EMT and cell mobility (Fig. 3A) [290]. Tensile and compressive stresses have distinct influences on YAP activity. Tensile stress increases YAP nuclear localization in transformed MCF-10A cells, while compressive stress mediates YAP cytoplasmic translocation via inhibiting F-actin formation in cervical cancer cells, or via reducing Rho activity and cortical contraction in fibrosarcoma cells [278, 291]. Further, 20-µm-width confinements force YAP to shuttle from the nucleus to the cytoplasm in bone osteosarcoma cells. Hyperosmotic stress induces YAP nuclear translocation through phase separation in U-2 OS cells and HEK293T cells, while high cell density suppresses YAP/TAZ activity, leading to contact inhibition in meningioma cells and breast cancer cells [292–294].

The tensed cytoskeletal structure is essential for force transmission from the cell membrane into the nucleus, and thus crucial for YAP nuclear localization. Direct force application to the nucleus can still induce YAP nuclear localization even when the cytoskeleton is disrupted, suggesting that nuclear mechanosensing is indispensable in YAP activation [56, 295]. However, there is controversy regarding the effect of nuclear mechanosensing on YAP. High nuclear envelope tension prevents nuclear deformation and force-induced YAP nuclear localization, while increasing nuclear pore size and permeability, which may promote YAP nuclear localization [296–298]. Additionally, nuclear mechanosensing can provide new feedback to cytoskeleton tension by regulating myosin II localization, which may further influence YAP nuclear localization. Since YAP lacks the nuclear localization sequence, how nuclear mechanotransduction translocates YAP into nuclei remains to be elucidated.

In contrast to biochemical cues, mechanical cues activate YAP in a Hippo-independent manner. Therefore, targeting YAP activity via Hippo signaling alone for cancer therapy needs to be revisited. Therapeutic intervention that can specifically target YAP/TAZ mechano-transduction or the downstream effectors promises to improve the precision of treatment.

#### Nucleus as a mechanosensor

In addition to mechanosensitive proteins, cell nucleus itself acts as a mechanosensor in response to mechanical stimulations. For example, forces directly applied to an isolated nucleus through nesprin-1 stiffen it within seconds via Lamin-A/C recruitment and emerin phosphorylation [299]. Force application to the nucleus is sufficient to drive YAP nuclear translocation by stretching nuclear pores even without the cytoskeleton. Further, when a cell passes through a confined space smaller than its nucleus, the nuclear envelope expands and unfolds, and the nuclear envelop tension increases, which induces calcium release from endoplasmic reticulum (ER) and triggers the recruitment of myosin II to the cell cortex [300]. Through this mechanism, cancer cells utilize their nuclei to measure the environmental confinement and adapt their behavior rapidly. The confinement in 3D stiff gels (~30 kPa) results in higher levels of nuclear deformation and mitotic defects than soft gels (5 kPa), which can be overcome by transformed but not normal cells possibly through the distinct nuclear mechanotransduction [69].

Nuclei can sense forces independent of the force transmission machinery, such as cytoskeleton and the linker of nucleoskeleton and cytoskeleton (LINC) complex. This is not contradictory to many other findings that cytoskeleton-based mechanotransduction is critical in response to mechanical cues applied on the cell surface. Instead, the cytoskeleton and associated proteins are essential in transmitting forces from the extracellular domain into the nucleus, but not required for nuclear mechanosensing. The concept of nucleus as a mechanosensor in tumor mechanobiology is under active investigation. First, when tumor cells migrate through the open-roof channels, the confinement from two side walls excludes YAP from the nuclei [301]. However, directly applying compressive forces to the nuclei leads to YAP nuclear accumulation. Further studies are required to explore whether the nucleus is mechanically heterogeneous and anisotropic, responding distinctly to forces exerted at different nuclear locations. Second, healthy and cancer cells exhibit different mechanosensitivity and distinct nuclear mechanics. As the nucleus is a critical mechanosensor, the difference in nuclear mechanosensing between healthy and cancer cells possibly contributes to the divergence of overall cell mechanosensitivity, which needs to be examined in the future.

#### Other mechanosensors/mechano-sensitive proteins

Besides the proteins described above, there are many other mechanosensors or mechano-sensitive proteins that are important in cancer. The representative is Caveolin-1 (CAV1), a membrane protein that directly connects to the actin cap and is mechanosensitive in many cell types [279]. Low shear stress increases the motility of breast cancer cells via CAV1-FAK and CAV1-ROCK signaling. Loss of CAV1 suppresses shear-mediated proliferation and induces cell death [302]. 20 mmHg of hydrostatic pressure promotes lung cancer cell migration through CAV-1-ERK signaling [106].

Transient receptor potential (TRP) channels interact with focal adhesion and cytoskeleton [303]. Among them, TRPV4 is a Ca2+ permeant channel and responsive to mechanical cues. Through TRPV4-PI3K/Akt signaling, tumor cells proliferate in weakly confined environments and undergo cell cycle arrest in highly confined environments [304]. In addition, knockdown of TRPV4 decreases breast cancer cell migration and invasion, while overexpressing TRPV4 facilitates actin depolymerization and decreases cellular stiffness [305]. The mechano-response of TRPV4 has been utilized to inhibit the abnormal angiogenesis formed by tumor endothelial cells (TECs). For example, TRPV4 is down-regulated in TECs compared to normal endothelial cells (NECs). On 370-Pa and 2280-Pa matrices, NECs show no difference in spreading area, while TECs show larger spreading area on stiffer substrates. Overexpressing TRPV4 in TECs inhibits abnormal angiogenesis, cell spreading, and migration [306]. Compared with normal fibroblasts, fibrosarcoma cells exhibit lower TRPM7 (another TRPP channel) and RhoA activity and are less sensitive to fluid shear stress. Their abilities of invasion and intravasation are TRPM7-dependent [32]. Breast cancer cells generate increasing levels of traction force on the substrates with elevating stiffness (from 1 to 30 kPa). Inhibition of TRPM7 decreases traction force and induces less mesenchymal phenotype [307].

### Summary

Tumor cells conduct mechanotransduction processes that interact with classical oncogenic signaling. Although significant progress has been made in cancer mechanobiology, several essential questions remain to be addressed.

First, mechanical cues can activate proteins following a cascade that is distinct from the classical biochemical pathway. For example, mechanical stimulation activates Rac independent of the classical upstream Src, the activity of which is essential in PDGF-induced Rac activation. In addition, mechanical cues can bypass the Hippo pathway and modulate YAP/TAZ activity. H1R, which belongs to GPCRs, is a mechanosensor in endothelial cells and its stretch-induced activation is independent of agonist induction. Therefore, it is possible that the mechanotransduction signaling intervenes oncogenic pathways independent of the traditional upstream signaling. As such, the anti-cancer drugs that only consider the classical oncogenic signaling should be revisited because their efficacy may be improved by co-targeting the independent mechanotransduction-activated signaling. Second, tumor cells encounter various mechanical cues at different stages of tumor progression. Living cells sense and respond to mechanical signals and keep the footprint of mechanical dosing history or mechanical memory for certain periods of time. Therefore, the mechanical dosing at earlier stages of metastasis may influence the mechano-sensitivity and -response of tumor cells to other mechanical cues at later stages. Hence, systematically investigating the influence of mechanical memory on tumor cell functions and the underlying mechanisms during tumor metastasis will be critical to elucidate the roles of mechanics in cancer and prevent the formation of metastatic tumors by targeting mechanical signals. Third, tumor cells at different stages may exhibit different levels of mechanosensitivity to various mechanical cues. It is still unknown whether these distinct responses are predetermined by the genotype and phenotype of tumor cells acquired in the primary tumor site or during metastasis. We hypothesize that tumor cells evolve and adjust mechanosensitivity to adapt to distinct mechanical contexts at different stages of metastasis. Fourth, it appears that the fate (winner or loser) of tumorous and healthy cells is partially determined by mechanical cell competition through the intercellular forces. It remains to be clarified whether the alterations in the mechanics of TME and tumor cells affect mechanical cell competition during tumor progression.

## Multiscale toolbox for the study of mechanobiology in cancer

### Tools for multiscale force measurement

**Fig. 4:**
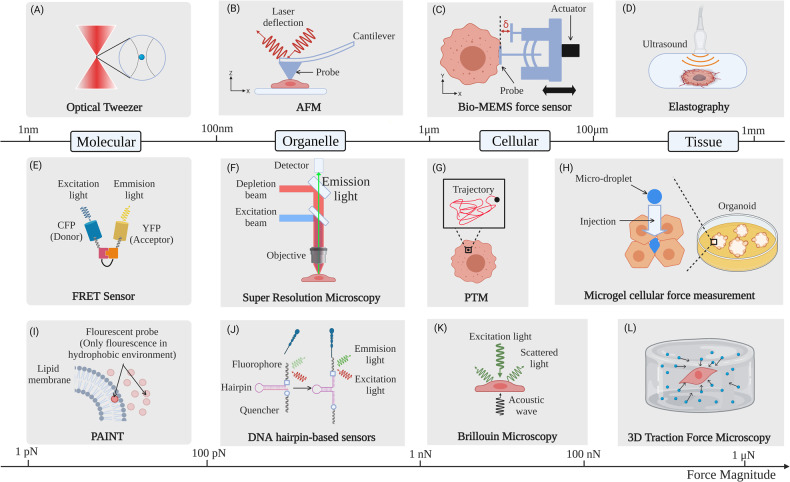
Biophysical tools for quantitative measurement, imaging, and manipulation of mechanical properties in living systems, across the molecular, cellular, and tissue scales. **A**–**D** Tools for application of mechanical force and measurement of mechanical properties (Section “Tools for multiscale force measurement”). **E**–**L** Tools for imaging and measurement of mechanical properties (Section “Tools for the in vivo measurement of tumor/tissue/cell stiffness”).

As mechanical forces critically influence living cells, technologies that enable mapping and applying forces are needed to deepen our understanding of tumor mechanics and design effective mechano-therapies [94, 306, 307]. Previous efforts in mechanobiology have invented a vast array of imaging (Table 5) and force measurement tools (Table 6) with broad spatial-temporal force ranges and resolutions at multiple scale [214, 308–310]. In this section, we highlight the representative force measurement/application techniques with the features of being nondestructive, quantitative, high-throughput, and long-term interrogations in cells and tissues (Fig. 4).

**Table 5:** Summary of bio-imaging tools’ characteristics.

Imaging tools		In vivo/ In vitro	*X-Y* Resolution	*Z* Resolution	Imaging Depth	Acquisition Speed	Refs
Confocal microscopy		In vitro/ in vivo	250 nm	700 nm	50–100 μm	30 FPS	[461, 462]
Two-photon microscopy		In vitro/ in vivo	500 nm	1500 nm	1mm	16 FPS	[463]
Infrared spectroscopy		In vitro/in vivo	2.6 μm	NA	4 cm	1000 FPS	[464, 465]
Super resolution microscopy	STED	In vitro/ in vivo	20–70 nm	110–150 nm	50–100 μm	20 FPS	[466]
	PALM/STORM	In vitro	20–50 nm	50 nm-4 μm (dependent on photon output)	200 nm	Minutes/ frame	[467]
Single cell MRI		In vivo	100 μm	200 μm	NA	NA	[468]
ExM		In vitro	25 nm	90 nm	NA	Single trial	[469]

**Table 6:** Tools for multi-scale force measurement in living systems.

Force measurement tools	In vitro/ in vivo	Object of measurement	Application scale	Measurement range	Resolution	Refs
FRET sensor and DNA hairpin-based sensors	In vitro/ in vivo	tensile force	molecular	1–20pN (10^-9 Newton)	1 pN	[310, 311]
Optical tweezer	In vitro/ in vivo	tensile force, viscoelastic property	molecular	0.1–100 pN	1 pN per 10 mW of laser power	[312]
Atomic force microscopy (AFM)	In vitro	tensile/compressive/adhesive force, viscoelastic property	molecular to tissue	fN (10^-15 Newton) to nN	femto-newton	[338]
Bio-MEMS force sensor	In vitro	tensile/compressive/adhesive force, viscoelastic property	molecular to tissue	1–250 nN	nN	[340]
Particle-tracking micro-rheology (PTM)	In vitro/ in vivo	viscoelastic property	Organelle	0–1900 Pa	Pa	[316]
3D traction force microscopy	In vitro	tensile force	Organelle to cellular	0–1 kPa	Pa	[320]
Elastic roundmicrogel method	In vitro/ in vivo	Solid stress	Cellular to tissue	Pa–kPa	Pa	[434]
Brillouin microscopy	In vitro/ in vivo	viscoelastic property	Cellular to tissue	kPa–GPa	Pa	[342]
Elastography	In vivo	viscoelastic property	Tissue	0–500 kPa	kPa	[344]

#### Force Measurement at the Molecular Level

Fluorescence resonance energy transfer (FRET) tension sensors and DNA hairpin-based sensors are two representative force measurement tools. FRET tension sensors can measure cell-generated tensions on single molecules with pico-Newton (10^-12^ N; pN) sensitivity. Specifically, this method inserts two fluorophores (donor and acceptor) connected by a mechanosensitive peptide linker into the biomolecules of interest. Subjected to tension, the length of the peptide linker alters, which influences the transfer of the emission light from the donor to the acceptor. The resulted change in the FRET efficiency is reflected by the spectral shift and fluorescence ratio between the donor and acceptor, providing a quantitative force estimate [311]. While being highly sensitive, however, FRET tension sensors are limited in only measuring a small force range, ~ 1–10pN. This is because larger forces exceed the endurable range of the linker so that the induced changes in FRET efficiency cannot be accurately detected [312]. FRET sensors embedded between two domains of vinculin have been used to quantify the tension within focal adhesion of breast cancer cells, showing the correlation between the magnitude of tension in focal adhesion and migration potential.

In a similar principle, DNA hairpin-based sensors utilize DNA hairpins as switches to generate altered fluorescence in response to the applied tension. The sensor consists of programmable stem-loop DNA hairpins that are conjugated between fluorophore-quencher pairs. When the hairpins are unfolded by a tension beyond a specific threshold, the quencher and fluorophore are separated so that the fluorescent intensity increases to report the force. Each sensor can be attached to a single receptor and the increase of the overall fluorescence is proportional to the number of receptors on which the force exceeds the threshold, enabling measuring the sub-cellular traction force.

Both types of sensors cannot determine the direction of the intracellular forces. Nevertheless, DNA-based sensors are extremely sensitive and have much higher force and spatial resolutions than most other tools (Table 6). In addition, DNA-based sensors do not interfere with cell biology and have a high signal-to-noise ratio due to their nearly binary response at the force threshold [313, 314].

#### Force Measurement at the Subcellular and Cellular Level

At the subcellular scale, the laser-ablation-based measurement can infer tension: after the ablation of a microfilament cable, the initial retraction velocity is proportional to the tension within the cable when the structure’s material properties are known [315, 316]. However, this technique is unable to measure the compressive loads. Particle-tracking micro-rheology (PTM) is an effective, contact-free, and noninvasive method. Fluorescent particles are injected into cell cytoplasm or nucleus and their mean-squared displacements are optically tracked, from which the viscoelasticity of the surrounding microenvironment can be derived using the theoretical models. PTM can measure the mechanical properties of highly localized subcellular regions inside 3D matrices or in vivo. Its spatial-temporal resolution depends on both the imaging system and particle properties (size, number, and distribution). PTM measurements show that the cytoplasmic viscoelasticity in malignant pancreas and breast epithelial adenocarcinoma cells is lower than that in their benign counterparts [317]. Combined with single-particle tracking and intravital microscopy, PTM can measure physical properties of cells in living organisms. Of note, both normal and cancer cells are less deformable in vivo than in 2D culture [318, 319]. Force-spectrum microscopy (FSM), a combination of PTM and optical tweezers, can measure cellular forces within living cells and provide insights into the cellular activities. For example, aggregate effects of motor proteins create random fluctuating force in cytoplasm. FSM measurement shows that benign cells have 3-fold lower force fluctuation than malignant cancer cells. For the in vivo imaging, because fluorescent particles need to be injected into target cells, PTM is only applicable to the implanted nonnative cells.

At the cellular scale, several noninvasive force measurement tools have been developed (Fig. 4), including geometry-based methods, 3D traction force microscopy, viscoelastic response to laser ablation and the novel use of molecular force sensors. First, multiple geometry-based cell force inference techniques, in which forces are inferred using curvilinear cell boundaries and geometry, have the advantage of enabling noninvasive and long-term measurements of dynamic processes. One such system, called CellFIT, estimates tension maps in both 2D and 3D conditions using image stacks in combination with mathematical models. It can determine the relative tensions of cell boundaries at triple junctions (points where the edges of three cells meet) using the angles between each cell boundary. Intracellular pressures can be determined using CellFIT through Laplace equations. Another geometry-based method relies on “fluid drop deformation”. Fluorescent oil microdroplets are coated with cell surface adhesion ligands and embedded between cells. The cell-generated forces can be quantified noninvasively from the microdroplet deformation [320]. One limitation of these geometry-based techniques is that they mostly provide the ratios between edge forces instead of absolute values. Second, 3D traction force microscopy can measure forces generated by single cells or tissue explants embedded in the 3D microenvironment. Matrix deformation is calculated from the 3D displacement of fluorescent beads and used to compute the distribution of cell-generated forces, given the known matrix material properties [321]. This tool has the desired ability to measure the dynamic traction forces of multicellular tissues. However, it cannot decouple cell-to-cell forces from cell-to-matrix forces. Third, after laser ablation of the cell-cell contact boundary, the distance retracted by the vertices of the ablated edge can infer the pre-existing tension within the ablated cell membrane [322]. Fourth, proper use of molecular force sensors can measure cellular forces when the measured object can represent the actual cellular connection. For example, tension gauge tether is used to measure the single-integrin-ligand-regulated cell-cell adhesion force (40 pN) during initial adhesion [323].

In addition, although with the capability of molecular scale measurement, optical tweezer and magnetic tweezer are widely used to measure force in cellular scale in cancer field. Optical tweezers utilize a highly focused laser beam to apply radiation pressure to the object at spatial and temporal resolutions of 0.1–2 nm and 0.1 ms, respectively (Fig. 4). The focused laser beam creates an optical trap because the difference between the refractive index of the medium and the object changes the momentum of light rays, generating the force in the range of 0.1–100 pN on the trapped object [313] (1pN per 10 mW of laser power). This technique has the benefits of low noise, high versatility, and a wide range of applications for force measurement, but is susceptible to optical and temperature perturbations, potential photodamage, and heating artifacts. Optical tweezers have been used to measure mechanical properties of cancer cells and potentially harvest CTCs [324–329]. Magnetic tweezers apply magnetic field to the magnetic particles that are attached to or within the cells, generating force in the range of 2–4 nN [315, 316, 330]. Compared to optical tweezer, magnetic tweezer can generates larger force range [299, 330, 331]. Recently, we used magnetic particles to directly apply force on the nucleus and validated the mechano-sensing capability of the neucleus [295].

#### Force Measurement at the Tissue Level

Multiple methods have been developed to measure IFP in tumor tissues in vivo. First, the micropore chamber method has been tested in rats and determined that the IFP is 4–5-fold higher in the tumor than that in the normal tissue [332]. Second, fluorescence recovery after photobleaching (FRAP) has been performed in rabbits to determine the IFP [333]. Third, the transducer-tipped catheter method places a transducer-embedded needle into the tumor core. The measured IFP is +3.6 mmHg in the mice with melanoma and reduced to −0.3 mmHg in the angiogenesis-related gene NG2-knockout mice [334]. The IFP is different in ventral and dorsal positions of cervical and pancreatic carcinoma xenografts. Fourth, magnetic resonance imaging (MRI) can measure IFP with the spatial and temporal resolution at 0.23 × 0.23 × 2.0 mm^3^ and 14-sec in patients and mice xenografts [335–337]. Another noninvasive method is ultrasound poro-elastography that evaluates the spatial-temporal distribution of IFP in a tumor [338]. The results measured by the last two methods show that the IFP is elevated in the tumor compared with the surrounding tissue and has little variation inside the tumor [337, 338]. Overall, new noninvasive tools with higher sensitivity are desired to evaluate the distribution of IFP within a tumor.

Several methods have been developed to measure solid stress in tumors (Table 7) [2]. First, the planar cutting method embeds tumor tissues in agarose gels, which are cut to allow the tissue to deform by releasing solid stress and elastic energy. The deformation profile of the cut plane is mapped to calculate mechanical stress through a mathematical model. The result shows that the core of breast and pancreatic tumor is under compression while the tumor periphery is under tension. Second, a slicing method enables estimating solid stress in small tumors. Tumor slices of 100–500 um thickness are cut and detached so that the solid stress can be released, causing the slices to bend and buckle as they expand in area. The change in surface area is used to compute solid stress. The measurement shows that solid stress increases when the tumor size increases and that elastic energy and tissue stiffness are not necessarily correlated. Third, the needle-biopsy method allows for in-situ quantification of solid stress. A hole with 1.5–3 mm in diameter is created in the tumor with rotating biopsy punches. As solid stress is released as a function of time, the hole deforms and changes in diameter, which can be converted to solid stress. This method is used to measure the solid stress in a murine brain tumor ex vivo, which reveals a maximum radial stress at 0.02 kPa in compression, as opposed to 0.1 kPa in compression in an in-situ case [2].

**Table 7:** Methods to measure solid stress within normal and tumor tissues.

Methods	Strengths	Limitations	Refs
Planar-cut method (in vitro)	• Map solid stress distribution across the whole single planar surface of one tumor • Can measure solid stress within tumors with diameter >1.5mm	• Cannot analyze tiny solid stress • Resolution: 0.1–10 kPa. • Only one-time measurement. • Only one plane is measured in each tumor.	[44]
Slice method (in vitro)	Highly sensitive (can measure solid stress within tumors with diameter>1mm and combine with ultrasound to measure deformation down to 20 µm)	Cannot differentiate the compressive from tensile solid stress because it’s an indirect method and depends on the expansion of surface area.	[79]
Needle biopsy method (in situ)	• Do not need to isolate the whole tumor for measurement. • Preserve the effect of the surrounding tissue on solid stress. • Can measure solid stress within tumors with diameter >1.5 mm.	• Less sensitive than the slice method • Resolution: around 0.02-0.1 kPa	[79]
Elastic round microgel method (in vivo)	• In vivo method to detect the cell-generated solid stress in living embryos. • Can distinguish compressive from tensile solid stress within tumors with diameter «1 mm.	• Fluorescent imaging methods have measuring depth of only ~ 200 µm due to light scattering and absorption in tissues. • Microgel cannot be delivered accurately, the solid stress can only be measured where droplets exist. • Too many droplets may impair tissue development.	[434]
Poroelastography method (in vivo)	noninvasive method to detect solid stress in the mouse model	The model assumes that the tumor is spherical and has constant mechanical properties (e.g., Young’s modulus).	[435]

Overall, each technique has its specific applications. Technologies with the ability for simultaneous measurement and manipulation of biomechanical forces will be extremely useful in the study of tumor mechanobiology.

### Tools for the in vivo measurement of tumor/tissue/cell stiffness

At the cellular and subcellular scales, cellular and nuclear stiffness can be measured by AFM, Bio-MEMS, optical stretcher, micropipette aspiration, and Brillouin microscopy (Fig. 4). AFM and Bio-MEMS biosensor are widely used to measure local cell stiffness or nuclear stiffness [339, 340]. Bulk cell stiffness can be measured by microfluidic devices and optical stretcher in which the magnitude of deformation indicates cell stiffness [341, 342]. Micropipette aspiration quantifies cell membrane tension based on the deformation controlled by micropipette diameter and suction pressure [343]. These techniques adopt different principles and mathematical models, which can be one of the reasons why the reported tumor cell stiffness shows large variations even within the same tumor type.

At the tissue scale, Brillouin microscopy is an accessible, noninvasive, and label-free optical tool for force measurement at a sub-micrometer resolution. Based on the effect of spontaneous acoustic waves on light scattering, this technique utilizes the frequency shift of the scattered light to determine the longitudinal modulus. Rigid/soft materials produce a high/low frequency shift. The limitations include the long measurement time (tens of minutes to hours) [344], which may introduce adverse phototoxicity and thermal effects on living tissues, and the weak signals of light scattering [345]. Brillouin microscopy has been used to identify tumor boundaries and elucidate the differences in elasticity between healthy tissues, melanoma tissues, and regressing tumors. Recent evidence using Brillouin microscopy shows that tumor cell nucleus softens and stays soft for over 24 hours after extravasation, indicating that this technique can be used for the stiffness measurement at the subcellular scale. Elastography, including ultrasound elastography (USE) and magnetic resonance elastography (MRE), can be used to noninvasively measure tissue stiffness in vivo. USE has two main subtypes, strain imaging (SI) and shear wave imaging (SWI). SI utilizes external ultrasound stimulation or internal physiologic motion under ultrasound to induce tissue deformation. The applied stress and the induced deformation can be measured to calculate Young’s modulus of the tissue. SWI utilizes ultrasound shear wave that propagates across the target tissue. Shear wave velocity can be measured by ultrasound probe to calculate the shear modulus of the tissue based on tissue density. USE can help clinical diagnosis of liver, breast, prostate, thyroid, brain, kidney and lymph node tumors combined with B-mode ultrasound [346]. For example, USE can distinguish F3/F4 liver fibrosis from F0/F1 stage, but not for diagnosis [347]. However, deep-tissue imaging by USE becomes less accurate due to the limited penetration of ultrasound (in centimeter scale with up to 100-μm spatial resolution) [348].

In MRE, mechanical vibration (excitation) at 50 – 500 Hz is applied on the target tissue to generate shear wave. Shear modulus (µ) can be calculated from the excitation frequency (ν), tissue density (ρ), and measured wavelength (λ) of the shear wave by $\mu ={{\rm{\nu }}}^{2}{{\rm{\lambda }}}^{2}{\rm{\rho }}$. Dynamic shear modulus images are obtained based on the imaging of the wavelength and this relation [349, 350]. Different from USE, MRE reports the whole shear modulus complex by a shear viscosity term [349–351]. Shear viscosity is related to the time-dependent loss of shear wave energy and mainly reflected by the attenuation of the waves as they travel through a medium. The resolution of MRE is typically 150–250 μm without noticeable depth limitation [352]. MRE shares similar applications as USE with comparable or better accuracy, especially in deep tissue imaging [352, 353]. MRE can map the stiffness of brain, liver and breast tumor tissue, characterize the tumor mechanical properties, and facilitate cancer diagnosis [9, 354, 355]. For example, in vivo MRE measurement shows that the stiffness of liver tissue positively correlated with histologic grade of fibrosis (~4 kPa and 10 kPa for fibrosis score at 2 and 14, respectively) and hepatocellular carcinoma is detected at high fibrosis score by biopsies [354]

Optical coherence tomography (OCT) elastography utilizes the light scattering properties and refractive index variations among different tissues to produce 2D/3D cross-sectional distribution of stiffness [356]. This noninvasive and label-free technique can be used in vitro, ex vivo, and in vivo and has the advantages of easy tissue-preparation/radiation-free, which are common challenges encountered by USE and MRE. However, OCT imaging/elastography is limited to shallow imaging depths of 1–2 mm for nontransparent tissues, due to optical scattering and absorption, and long acquisition durations [357]. OCT generally has a resolution of 1–20 µm in the transverse and axial directions [358]. OCT elastography can distinguish tumor tissues from normal tissues based on the stiffness difference when integrated with small probes and catheters for the examination of internal organs [359].

Currently, computer vision techniques applied on clinical breast cancer images and analysis of cancer biomarkers are combined to automatically predict cancer risk and prognosis from images [360, 361]. Artificial intelligence (AI) automates the processes of cancer detection and treatment evaluation, eliminating the need for intensive human interpretation of images and stiffness results [362, 363]. However, all tools described in this review have not been commonly used in the clinics, likely due to their limitations and difficulties for in vivo applications.

### Organoid

Organoids are 3D miniaturized tissue structures that self-assemble and function in vitro [364]. Tumor organoids are usually formed by patient-derived tumor cells and CAFs that secrete patterning factors, e.g., EGF and FGF, in a 3D pathologically relevant microenvironment (Fig. 4), including stroma and immune cells [365]. Despite the lack of vasculature, this method has generated tumor organoids in several cancer types, such as colorectal (CRC), gastrointestinal, pancreatic, prostate, breast, and liver cancer. Recently, vasculature has been developed in breast and pancreatic tumor organoids [366, 367].

Recent research incorporates biomechanical factors into tumor organoids, such as microenvironment stiffness, shear perfusion stress, and cell mechanics (Table 8). As discussed in Section “Mechanical alterations in primary tumore tissue,” tumor tissues exhibit altered structure and higher stiffness than their healthy counterparts. Stiff gelatin-phenol hydrogels (shear modulus: 14.3 kPa) grow larger CRC organoids with higher expression of ITGA6 than soft gels (shear modulus: 2.6 kPa). However, gels with stiffness beyond 22.3 kPa have minimal effect on the volume of tumor organoids and ITGA6 expression [368]. Another study reports that stiff CRC organoids lose the mesenchymal phenotype but upregulate stemness markers, while soft organoids are more resistant to chemotherapy [369]. One potential mechanism may be that upon chemotherapy, stiff matrix activates JNK signaling to induce cell apoptosis while the soft matrix activates NF-kB signaling to suppress JNK signaling [370]. For pancreatic cancer organoids, when matrix stiffness gradually increases (1.4, 3.1, 8.2, and 20.5 kPa), YAP translocates into the nucleus, promoting the organoid growth [371]. Except matrix stiffness, lung cancer organoids that are developed in a microfluidic device upregulate CSC markers under dynamic perfusion compared to static conditions [372].

**Table 8:** Organoid models utilized in biomechanics-related research.

Type of tumor organoid	Matrix type	Tumor cell type and sources	Other types of cells	Organoid size	Mechanical stimulation	Influences on tumor progression	Refs
Murine pancreatic cancer organoids (mPCOs); Human pancreatic ductal organoids (hPDO)	PEG based hydrogel (with FN, Col, and BM -mimicking peptide)	From mice model; From patient	murine pancreatic fibroblasts and macrophages	100 µm	Increased matrix stiffness (1.4, 3.1, 8.2, and 20.5 kPa)	Stiff environments promote YAP nuclear translocation. Organoids grow better in stiffer hydrogels, despite that the optimal stiffness vary for organoids whose cells are derived from different mice tumor tissues.	[371]
Colorectal cancer organoid	Gelatin-phenol hydrogel	From patient-derived xenograft	NA	100 µm	Increased matrix stiffness (2.6, 14.3,22.3, and 34 kPa)	The optimized stiffness for tumor organoids to grow is 14.3 kPa	[368]
Lung cancer organoid	Matrigel	From patient	NA	About 200 µm	2–5 ml/day fluid perfusion	CSC-related markers are upregulated under perfusion-mediated shear flow.	[372]
Colorectal cancer organoid	Type-1 collagen	CRC cell line	Myofibroblast	Several mm	Increased tissue stiffness (10 to 50 kPa)	In stiffer organoids, the mesenchymal phenotype of tumor cells is inhibited but the expression of stemness marker is upregulated, while tumor cells in soft organoids are more resistant to chemotherapy	[369]
Breast cancer organoid	Alginate–Matrigel	Mammary epithelial cell line	NA	About 50 µm	Alteration of cell mechanics	Tumor cells in the organoid periphery show lower cellular stiffness, larger cellular and nuclear volume as well as higher motility compared with those in the organoid core	[68]
Breast cancer organoid	BM–conjugated PA gels	Breast cancer cell lines	NA	About 100 µm	Increased matrix stiffness (0.15 to 5 kPa)	Tumor organoids cultured in soft environments are more resistant to chemotherapy or irradiation than those cultured in stiff environments.	[370]

Tumor organoids possess unique advantages compared with the 2D/3D cultures and patient-derived tumor xenografts (PDTXs). First, in 2D culturing method, only a subpopulation of tumor cells can be selected for sustainable proliferation. Tumor cells cultured in 3D ECM can better mimic the microenvironment compared with 2D cell culturing [373]. However, whether and to what extent this method maintains the bona fide properties of in vivo tumors remain unclear. PDTXs are a widely used in vivo model that can mimic tumor progression. However, to control various factors in vivo is challenging [374]. Translating the pre-clinical findings derived from PDTXs into clinics remains challenging, because of the physiological difference between mice and humans [375]. In contrast, tumor organoids recapitulate the intratumoral heterogeneity of in vivo human tumors and maintain genetic and phenotypic features in vitro for months. Co-culturing human immune cells with tumor organoids can better mimic the in vivo tumor immune microenvironment [376]. Second, the organoid model can better mimic TME because mechanical factors can be incorporated in a controlled manner, mimicking the tumor tissue stiffening and the accumulation of solid stress. Overall, this new method provides a pre-clinical tumor model for cancer research and drug development, despite that it requires time-and resource-consumable experiments, which limits its wider applications.

### Future tools for tumor mechanobiology and beyond

Current technologies described in Sections Tools for multiscale force measurement, Tools for the in vivo measurement of tumor/tissue/cell stiffness, and Organoid (Fig. 4) have been utilized in tumor mechanobiology to provide extensive knowledge about the roles of mechanical forces in tumor growth and progression. Nevertheless, our understanding of the influence of mechanics is still in the infancy stage mainly because of the complexity of cancer and the lack of more sophisticated technologies in mechanobiology. Genetic and phenotypic heterogeneity is a hallmark of cancer so that the biomarker proteins that are utilized for cancer diagnosis and treatment are usually specific for one cancer type/subtype at a specific stage of tumor progression. This challenges the diagnosis precision, treatment efficacy, and the generic applications of these biomarkers across different cancer stages and cancer types. In comparison, as we reviewed in Sections Tools for the in vivo measurement of tumor/tissue/cell stiffness and Tumor cell mechanics, cancer cells originating from most organs are softer than their healthy counterparts, and tumor tissues are usually stiffer than healthy organs. We propose that these biophysical signatures of tumor cells and tissues may be used as generic mechanical hallmarks for both diagnosis and therapy, outperforming biomarker-based approaches. Towards these goals, it is imperative to develop prospective mechanobiology tools with the capability to enable in vivo measurement of stiffness at both single-cell and tissue scales in a safe, noninvasive, deep-tissue-penetrating, and long-term fashion. Such tools can scan the patients regularly to detect the atypical singularity, i.e., discontinuous value of cell and tissue mechanical stiffness. Combined with liquid biopsy, the efficacy and accuracy of early tumor detection and metastasis prediction can be enhanced.

Further, motile cancer cells form invadopodia and generate active contractile forces at the tumor front. We propose that cell-generated active force may serve as another mechanical marker to distinguish invasive cancer cells from the surrounding benign and healthy counterparts. Recent technologies that quantitatively map cell contractile forces in 2D/3D conditions are mostly applicable for the cells cultured on synthetic gels or micro-post array. To date, no method allows the traction measurement of single cells within 3D tissues. We believe that new technologies that enable in vivo cellular force mapping will be beneficial for early tumor detection and diagnosis. In addition, these technologies may guide the pharmacological treatments to the targeted cancerous cells or local tissues and evaluate the efficacy before it is too late, i.e., distant dissemination.

## Mechano-medicine and mechanotherapy

Multiple therapeutic modalities have been developed to treat cancer, including surgical resection, radiotherapy, chemotherapy, and immunotherapy [377, 378]. However, a typical limitation of them is poor specificity: they often target both cancer and healthy cells due to the lack of specific markers solely for cancer cells. Immunotherapy has improved performance in targeting specificity, but could still act against healthy cells due to overstimulated or misdirected immune responses [379].

Cancer biomarkers, such as CD123, CLL-1, and PD-1, have been identified to specifically target tumor cells in limited conditions [380]. However, most of these biomarkers can only recognize one specific cancer type or subtype [381] and usually evolve during tumor progression. Thus, identifying universal markers for different cancer types at various stages is critical for targeted cancer treatments. In preceding sections, we have summarized the distinct mechanical signatures of TME, tumor tissues, and tumor cells in comparison to their healthy counterparts. Many of these mechanical (e.g., stiffness) and biophysical (e.g., pHe) signatures appear to be generic across various cancer types [382], providing potential targets for cancer mechano-diagnosis and mechanotherapy. In this section, we categorize four major groups of mechanomedicine that target these mechanical features and mechanotransduction signaling and discuss promising ideas of mechano-therapeutics (Table 9).

**Table 9:** Potential mechanomedicine and strategies for cancer therapy by targeting different mechanical signatures of tumor.

Strategies	Mechanomedicine to achieve	Therapeutic effects demonstrated in vivo	Refs
Target tumor tissue stiffness	Reduce tissue stiffness by: • reducing collagen crosslinking • inhibiting CAF activity • inhibiting fibroblast contraction	• Impede tumor progression • Suppress lung and liver metastasis • Improve chemotherapy	[4, 53, 385–388, 391, 392]
	Utilize tumor-specific tissue stiffness by: • employing YAP/TAZ	• Selectively identify and treat tumor tissues with high ECM stiffness	[280]
Target tumor-specific mechanotransduction pathways	Restore cell rigidity sensing by: • restoring normal levels of cytoskeletal proteins • increasing myosin-dependent cell contractility • activating mechanosensitive channel TRPV4	• Inhibit tumor formation • Reduce tumor cell invasion capacity and prolong survival time • Normalize tumor angiogenesis	[144, 305, 393]
	Impede mechano-signaling pathway: • ERK • YAP/TAZ • FAK	• Suppress stiffness-enhanced tumor growth • Stop transformation of primary cells? • Re-sensitize tumor cells to drug treatment	[3, 20, 374, 395]
	Utilize mechano-response of tumor cells by: • inhibiting ESCRT III • employing Piezo1 under mechanical stretch • employing Piezo1 under ultrasound	• Trigger tumor cells to undergo nuclear envelope rupture and DNA damage? • Specifically kill cancer cells and reduce tumor growth? • Enable accurate control of CAR-receptor T cell activation by ultrasound	[191, 216, 402–404]
Target the low stiffness of tumor cells and nuclei	Increase cell stiffness by: • enhancing the cortical localization of myosin IIC • elevating action polymerization	• Reduce metastases • Impede extravasation • Improve T cell immunotherapy	[123, 129, 145, 405]
	Utilize tumor cell softening to: • facilitate uptake of T-MPs and nanoparticles	• Reduce drug resistance	[406, 407]
	Increase nucleus stiffness by: • overexpressing Δ50 lamin A	• Suppress cancer cell invasion	[123, 410]
Target the mechanics of tumor vasculature	Release the pressure on tumor blood vessels by: • down-regulating hyaluronic acid and type I collagen of ECM • decreasing the recruitment of CAFs • reducing the interstitial fluid pressure (IFP)	• Improve efficacy of drug delivery and reduce hypoxia • Inhibit tumor growth • Induce tumor cell necrosis • Provide entry for immune cells	[91, 411–424]
	Utilize shear flow in blood vessels to: • facilitate medical treatment for CTCs via ES/TRAIL liposomes	• Increase apoptosis of CTCs and functionalize leukocytes	[425]

### Targeting tumor tissue stiffness

Multiple types of cancer, including breast, colorectal, prostate, and liver cancer, show stiffened tumor tissues, suggesting that high tissue stiffness is a mechanical hallmark of cancer [383–386]. Several pioneering attempts have harnessed this unique hallmark for cancer treatment. For example, reducing LOX-mediated collagen crosslinking prevents tissue fibrosis and stiffening, decreases FAKpY397, and impedes breast tumor progression in vivo [53]. To date, several pharmaceutical drugs targeting the mechanics of TME are undergoing clinical trials [4, 387]. Fresolimumab, an antibody against TGF-β, is undergoing the examination of its anti-fibrotic effects, in combination with radiotherapy for the treatment of stage IA-IB nonsmall cell lung cancer [388]. M7824, a fusion protein blocking both PD-L1 and TGF-β pathways, reduces the expression of α-smooth muscle actin (α-SMA) in mouse tumors. M7824-treated tumors exhibit thinner fibers and lower density in the collagen network compared to isotype control. M7824 treatment also suppresses spontaneous breast cancer metastasis in a mouse model [389]. The PHD2-inhibitor DMOG inhibits CAF activity, reduces tumor stiffness, and suppresses CAF-mediated lung and liver metastasis in a mouse breast tumor model [390].

In addition, elevated ECM protein (e.g., collagen, fibronectin, laminin) expression and ECM stiffness confer drug resistance in several types of cancer, raising the possibility that targeting the stiffness and composition of ECM may overcome chemoresistance [391, 392]. Inhibitors of the renin-angiotensin system, such as losartan and ramipril, have been used to reduce fibroblast contraction and remodel ECM composition, thereby decreasing tissue stromal stiffness in liver metastases. The softened tissue increases the anti-angiogenic effect of bevacizumab and effectively prolongs patient survival by inhibiting the proliferation of endothelial cells and improving drug efficacy [393].

Targeting tumor tissue mechanical properties can also facilitate the delivery of CRISPR/Cas system and enhance the gene-editing efficacy for cancer therapy. Lipid nanoparticle (LNP)-delivered FAK siRNA and CRISPER-PD-L1 enhance gene editing by reducing tumor ECM stiffness in liver cancer, therefore inhibiting in vivo tumor growth and extending survival of tumor-bearing mice [394].

The stiff tumor-specific microenvironments have been exploited for cancer therapies. For example, YAP/TAZ signaling is utilized to establish a mechanoresponsive cell system (MRCS). When MRCS encounters breast cancer metastases that have increased ECM stiffness, YAP/TAZ translocate into the nucleus to activate a mechano-sensitive promoter that drives the transcription of a downstream gene cytosine deaminase (CD). Consequently, CD converts the nontoxic prodrug 5-FC into the cytotoxic 5-FU only at the stiff metastasis sites. The system specifically targets stiff tumor tissues, while leaving soft healthy tissues intact, thereby limiting off-target toxicity. It provides an adaptive platform to selectively identify and treat cancer metastasis by targeting altered biophysical properties in tumor sites in vivo [281].

### Targeting tumor-specific mechanotransduction pathways

Aberrant mechanotransduction signaling in tumor cells could be targeted to potentially reduce malignancy and eliminate tumor cells by re-normalizing the cellular mechano-responses. Multiple types of cancer cells have much lower rigidity-sensing ability than their healthy counterparts, due to the lack of contractile units (CUs), including cytoskeletal proteins (Myosin IIA and tropomyosin 2.1 (Tpm 2.1)), and kinases (EGFR, HER2 and ROR2) [395]. Multiple studies have shown direct evidence that restoring mechanosensitivity of cancer cells impedes tumor growth. Restoration of rigidity sensing, by re-expressing cytoskeletal proteins, blocks colony formation of cancer cells in soft agar and inhibits tumor formation in vivo [395]. Overexpression of a constitutively active (CA) mutant of RhoA in GBM TICs restores their mechanosensitivity to ECM stiffness, retards cell motility on soft ECMs by increasing myosin-dependent cell contractility, and suppresses tumor invasion in vivo [144]. Overexpression of mechanosensitive ion channel TRPV4 restores the mechanosensitivity to substrate rigidity of tumor endothelial cells (TECs) and reduces their migration. Pharmacological activation of TRPV4 by GSK1016790A in TECs normalizes tube formation in vitro and tumor angiogenesis in vivo, which reduces tumor growth in combination with cisplatin [306]. These results inspire the development of new cancer therapies to inhibit cancer migration and invasion, potentially by targeting the mechano-sensitivity of tumor and stromal cells towards their mechanical microenvironment.

Mechanotransduction pathways that are critical in tumor progression provide new targets for cancer therapy. Stiff gels promote the growth of tumors generated by heat-treated residual HCC cells in nude mice, which is associated with stiffness-dependent ERK phosphorylation. Treatment combining vitamin K1 and sorafenib decreases ERK phosphorylation and suppresses the stiffness-mediated growth of residual HCC in vivo [396]. Stiff ECM-mediated YAP/TAZ are required for the receptor-tyrosine-kinase (RTK)/Ras-induced transformation of healthy cells into tumor precursors [22]. Hence, targeting the mechanosensitive YAP/TAZ signaling raises the possibility of reducing oncogene-induced tumor initiation. Transmission of mechanical force from minority taxol-resistant cancer cells to the majority taxol-sensitive cancer cells elevates their contraction, adhesion strength, and drug resistance. Reducing the force transmission by a FAK inhibitor VS-4718 re-sensitizes tumor cells to taxol in vitro and in vivo, leading to increased therapeutic efficacy [397]. Several drugs and biological agents that target tumor mechanotransduction pathways have been approved by the FDA or are undergoing clinical trials [4].

The responses of tumor cells to TME have also been utilized to potentiate new mechano-medicines. During migration through confined space, the nuclear envelopes (NE) of tumor cells rupture, leading to chromatin protrusions, nuclear fragmentation, and DNA damage. Endosomal sorting complexes required for transport III (ESCRT III) proteins are recruited for restoration of NE integrity. Inhibiting both ESCRT III-mediated NE repair and ataxia telangiectasia mutated (ATM) kinase-mediated DNA damage repair promotes cell death after NE rupture [191], which can be utilized to kill disseminated tumor cells during their penetration through dense tumor ECM and intra/extravasation processes. In another study, growth-induced compression causes NE rupture and chronic DNA damage, leading to increased invasiveness of human breast cancer cells. Silencing TREX1 reduces collagen degradation in vitro and tumor invasion in vivo, suggesting the potential in inhibiting the compression-induced invasion [193]. These results suggest that cautions are required to target mechanically confined tumor cells by inhibiting NE and DNA repair, because the surviving tumor cells may become more invasive.

Further, mechanical stretch has been demonstrated to kill cancer cells but promote normal cell growth in vitro via mechano-sensitive Piezo1-mediated calcium signaling [216]. In another study, body stretching reduces tumor growth in vivo by activating immune responses [216, 398]. Indeed, physical exercise increases tumor blood flow and reduces tumor hypoxia, and the resultant high blood shear stress can kill more CTCs [399–401]. Transient compression can revert the phenotypes of malignant breast cells and normalize the growth and development of tumor colonies [402]. Nevertheless, at the early developmental stage of mouse colon tumor, the chronic mechanical pressure exerted by tumor growth activates the tumorigenic β-catenin pathway in the surrounding healthy epithelial cells to form tumorous aberrant crypt foci [81]. In vitro studies of human-organ-on-chip models reveal that mechanical breathing motions inhibit tumor cell growth and invasion but increase drug resistance in the orthotopic lung cancer model [402, 403]. Thus, two layers of consideration are necessary to harness mechanical stimuli for cancer treatment: (1) choose suitable force dosages to leverage beneficial effects while maximally bypassing side effects for patients, and (2) combine mechanotherapy and chemotherapy to avoid contradictory effects with each other.

A recent study shows that ultrasound wave specifically causes apoptosis of tumor cells, by inducing Piezo1-regulated calcium influx and activating a calpain-dependent mitochondrial pathway [404]. In another study, transformed CAR-T cells have been engineered to express the mechanosensitive Piezo1 channel and the genetic transducer. This molecular suite converts ultrasonic-wave-produced mechanical disturbance into pre-programed genetic activities to control spatial-temporal activation of CAR-T cells, guiding them to kill the targeted tumor cells in vivo [405]. Further, focused ultrasound can reversibly and precisely control the anti-cancer functions of CAR-T cells to suppress tumor growth [406]. These results potentiate more controllable and less invasive cancer mechano-therapies.

### Targeting the low stiffness of tumor cells and nuclei

Tumor cell stiffness is often low and inversely correlated with their malignancy, raising the possibility of treating cancer specifically by targeting their low stiffness. A drug 4-hydroxyacetophenone (4-HAP) has been synthesized to increase cortical tension of tumor cells and decrease cell deformability by enhancing the cortical localization of myosin IIC (MYH14). 4-HAP treatment reduces the invasion and migration of both invasive pancreatic/colon cancer cells in vitro and metastatic liver cancer cells in vivo [145, 407]. Another drug is Jasplakinolide (Jasp) that increases the stiffness of TRCs by promoting actin polymerization. The Jasp administration impedes the transmigration of cancer cells in a 3D matrix and the extravasation of TRCs in zebrafish vasculature [129]. Soft TRCs escape from cytotoxic T lymphocytes (CTL)-mediated killing via preventing CTL-released perforin from drilling pores through cell membrane. Jasp treatment increases the cell stiffness and the generation of perforin-caused pores on the membrane, and thus increases CTL-mediated killing of TRCs by more than 2-fold in the mouse model, which improves the efficacy of immunotherapy [123].

Further, malignant tumor cells or CSCs are highly tumorigenic, drug resistant, and much softer than less malignant tumor cells [37, 408, 409]. Recent studies suggest that cell softness might be a more specific marker of tumorigenic and metastatic tumor cells than traditional cell surface markers (such as CD133 and ALDH) [122]. Investigating whether low cellular stiffness dictates tumor cell malignancy is of increasing interest because such studies may lead to the development of novel mechano-medicines for cancer therapy by stiffening malignant cells or CSCs. However, the drug resistance ability of several types of cancer cells has been reported to be positively correlated with their cell stiffness, raising the possibility that stiffening these cancer cells might deteriorate the efficacy of chemotherapy [338, 410]. Hence, cautions must be taken when targeting cancer cell softness alone for cancer therapy.

In parallel to the efforts in stiffening tumor cells, low stiffness of tumor cells has been exploited to improve the efficacy of chemotherapy. Being highly soft (deformable), TRCs can efficiently take up tumor cell-derived microparticles (T-MPs) that contain anti-tumor drugs through an unknown but nonphagocytosis pathway. Drug-carrying T-MPs effectively eliminate TRCs in vivo and prolong the survival of tumor-bearing mice, suggesting a novel approach against drug resistance [408]. Our recent evidence shows that the softness of breast CSCs facilitates the uptake of nitrogen-doped graphene quantum dots conjugated with chemotherapy drugs partially by activating clathrin-/caveolae-mediated endocytosis, promoting the specific elimination of these soft CSCs [411]. These data suggest a new route for targeted drug delivery into malignant and soft tumor cells.

The softness of tumor cell nuclei facilitates tumor cell invasion in dense tumor stroma and the transmigration through narrow endothelial cell junctions during intra- and extra-vasation. Tumor cell nuclei become softened during and after trans-endothelial migration [126]. Stiffening the nuclei through recombinant overexpression of Δ50 lamin A suppresses the invasion of melanoma cells [412]. These data suggest that targeting the softness of tumor cell nuclei holds the potential to suppress cancer metastasis.

### Targeting the mechanics of tumor vasculature

To renormalize the tumor vasculature that is tortuous and leaky, multiple drugs have been developed to reduce the mechanical pressure applied on tumor blood vessels, enhance drug delivery, and reduce hypoxia. Anti-fibrotic drug pirfenidone (PFD) is packed in an imine-based COF (COF_TTA-DHTA_) and accompanied by the decoration of poly(lactic-co-glycolic-acid)-poly (ethylene glycol) (PLGA-PEG) to fabricate PFD@COF_TTA-DHTA_@PLGA-PEG (PCPP). PCPP selectively accumulates in the tumor area and releases PFD in situ to down-regulate hyaluronic acid and type I collagen in the ECM. This regimen reduces the mechanical pressure on tumor microvasculature, doubles the effective area density of blood vessels, and improves the oxygen supply of tumors in vivo, thereby improving the therapeutic effects [413]. Another angiotensin inhibitor losartan decreases the solid stress and decompresses blood vessels in the mouse model of breast and pancreatic tumors by reducing stromal collagen and hyaluronan production, facilitating the delivery of both drugs and oxygen into the tumor [93]. Similarly, anti-fibrotic drugs Tranilast and PFD alleviate solid stress by reducing collagen and hyaluronan in tumor tissues, which decompresses vasculature and improves the efficacy of chemotherapy and nanomedicine [414–416].

In addition, CXCL12/CXCR4 signaling is found to promote fibrosis in both the primary and metastatic TME of breast cancer. Inhibition of CXCR4 by AMD3100 in a mouse model decreases the recruitment of activated CAFs into the TME, and further reduces solid stress. AMD3100 treatment decompresses tumor blood vessels and reduces hypoxia [417]. Recent studies show that the compression of vasculature can be decreased to facilitate the delivery of photosensitizers (PSs) and augment the efficacy of photodynamic therapy (PDT). The TGF-β inhibitor LY2157299 reduces collagen deposition and releases mechanical pressure on tumor blood vessels. The process increases the penetration of hydroxyethyl starch–chlorin e6 conjugate self-assembled nanoparticles (HES–Ce6 NPs), which function as PSs, into tumor tissues, and enhance the efficacy of PDT [418]. Mechanotherapy that reduces the solid stress in tumors has been combined with chemotherapy and is currently undergoing a phase-II clinical trial [419]. These results potentiate novel mechanomedicine to enhance the delivery of drugs, nanoparticles, and oxygen into the tumor by reducing the solid stress on vasculature. However, in vitro studies of tumor spheroids show that solid stress of 37.5–120 mmHg (exceeding blood pressure in tumor vessels) inhibits growth, suppresses proliferation, and induces apoptosis of cancer cells [86–88]. Thus, consideration should be taken for cancer patients who have high solid stress (37.5–120 mmHg), since reducing the stress can have adverse effects and facilitate tumor growth.

Prostaglandin E1 (PGE1) reduces IFP and increases capillary‐to‐interstitium uptake of ^51^Cr‐EDTA in tumors, which enhances the delivery of chemotherapy drug into the tumor to inhibit the growth of rat colonic carcinoma and mammary carcinoma [420]. The vascular disrupting agent ZD6126 decreases blood flow, oxygenation and IFP, inducing cell necrosis in the core of murine fibrosarcoma and human cervical carcinoma [421]. Another vascular disrupting agent combretastatin-A4 disodium phosphate (CA4DP) decreases tumor perfusion and reduces tumor IFP [422]. Sterically stabilized liposome SSL-IMA impedes fibroblast proliferation and vasculature formation through inhibition of PDGFR-beta by blocking the phosphorylation of the receptors. The consequent effects further decrease IFP by 42.37%, which can increase the anti-tumor efficacy of doxorubicin in melanoma [423]. Vascular endothelial growth factor (VEGF) inhibitor decreases microvascular density and tumor IFP, facilitates drug penetration, and delays tumor growth [424]. High-frequency ultrasound can influence blood perfusion and decrease tumor IFP (a) from 17.7 mmHg to 12.9 mm Hg (3MPa-USMB) and (b) from 15.3 mmHg to 9.8 mmHg (5MPa-USMB) [425]. In addition, a “nano-lymphatic” system is constructed to decrease the volume of the tumor interstitial fluid and reduce IFP by 37.89% in the tumor tissue in vivo, based on g-C_3_N_4_-mediated, light-activated water splitting process. It enhances blood perfusion and drug penetration to the tumor center, therefore reducing tumor hypoxia [426].

Moreover, mechanical cues in vasculature have been utilized to eliminate CTCs. Because CTCs adhesively interact with the endothelium via E-selectin (ES) in shear flow, liposomes conjugated with both ES and tumor necrosis factor (TNF)-related apoptosis-inducing ligand (TRAIL) are utilized to kill cancer cells under circulatory shear stress, by binding death receptors 4 and 5 on the cell surface. Importantly, ES/TRAIL liposomes increase apoptosis of circulating COLO 205 cells before and after lodging into mouse lung and functionalize leukocytes to target and kill CTCs in circulation, without inducing notable leukocyte death [427].

In addition to these four major groups of mechanomedicine (Table 9), new mechanotherapeutics need to be developed against cancer from other biomechanical perspectives. On one hand, mechanical deformation by squeezing cells through microfluidic channels transiently disrupts the cell membrane and facilitates intracellular delivery of macromolecules, which may be utilized to enhance the delivery of chemotherapy drugs [428, 429]. Recent studies find that the microparticles (MPs) secreted by TRCs are softer than those secreted by conventional tumor cells. These soft MPs show enhanced penetration through blood-vessel walls and accumulation in tumor tissues, suggesting a new mechanomedicine strategy to improve drug delivery efficiency [430]. These findings potentiate the development of novel mechanical methods for drug delivery into tumor cells. On the other hand, leveraging the durotaxis of fibroblasts, a recent study demonstrates that inhibition of microtubule dynamics can effectively suppress fibroblast differentiation on stiffened matrices and the resultant fibrosis of the lungs, providing a new target for anti-fibrotic therapy [431]. In vitro models of liver fibrosis reveal that mechanotransduction-regulated angiogenesis induces fibrogenesis in a stage-dependent manner [432, 433]. In addition, recent studies in muscle regeneration of mice show that appropriate mechanical stimulations improve muscle healing and regeneration by facilitating neutrophil clearance and reducing proinflammatory cytokines/chemokines [434]. This data links mechanotherapy and immunotherapy and potentially can be applied to tumor treatments. Together, these advances enlighten unconventional targets and strategies against cancer-associated fibrosis through the intervention of the mechanotransduction process.

## Summary and outlook

Due to the page limits, some relevant excellent literature may not be cited here. This review has not covered the rapidly developing fields such as the roles of exosomes in primary tumor and premetastatic niche, the mechanobiological function of tumor-associated macrophages in primary lesion, neo-antigen-based immuno-editing/immunotherapies that target a subpopulation of cancer cells, tumor microbiome, and tumor metabolic therapy that targets glucose and glutamine. These are all highly relevant to tumor biophysics discussed in this review and each deserves to be the subject of additional reviews.
